# Untangling the mess of CGRP levels as a migraine biomarker: an in-depth literature review and analysis of our experimental experience

**DOI:** 10.1186/s10194-024-01769-4

**Published:** 2024-04-29

**Authors:** Gabriel Gárate, Julio Pascual, Marta Pascual-Mato, Jorge Madera, María Muñoz-San Martín, Vicente González-Quintanilla

**Affiliations:** Instituto de Investigación Marqués de Valdecilla (IDIVAL), Hospital Universitario Marqués de Valdecilla & Universidad de Cantabria, Santander, Spain

**Keywords:** ELISA, Exercise, CGRP, Half-life, Method, Migraine, Storage

## Abstract

**Background:**

Calcitonin gene-related peptide (CGRP) is the most promising candidate to become the first migraine biomarker. However, literature shows clashing results and suggests a methodological source for such discrepancies. We aimed to investigate some of these methodological factors to evaluate the actual role of CGRP as biomarker.

**Methods:**

Previous to the experimental part, we performed a literature review of articles measuring CGRP in migraine patients. Using our 399 bio-bank sera samples, we performed a series of experiments to test the validity of different ELISA kits employed, time of sample processing, long-term storage, sampling in rest or after moderate exercise. Analysis of in-house data was performed to analyse average levels of the peptide and the effect of sex and age.

**Results:**

Literature review shows the high variability in terms of study design, determination methods, results and conclusions obtained by studies including CGRP determinations in migraine patients. CGRP measurements depends on the method and specific kit employed, also on the isoform detected, showing completely different ranges of concentrations. Alpha-CGRP and beta-CGRP had median with IQR levels of 37.5 (28.2–54.4) and 4.6 (2.4–6.4)pg/mL, respectively. CGRP content is preserved in serum within the 24 first hours when samples are stored at 4°C after clotting and immediate centrifugation. Storages at -80°C of more than 6 months result in a decrease in CGRP levels. Moderate exercise prior to blood extraction does not modulate the concentration of the peptide. Age positively correlates with beta-CGRP content and men have higher alpha-CGRP levels than women.

**Conclusions:**

We present valuable information for CGRP measurements in serum. ELISA kit suitability should be tested prior to the experiments. Alpha and beta-CGRP levels should be analysed separately as they can show different behaviours even within the same condition. Samples can be processed in a 24-h window if they have been kept in 4°C and should not be stored for more than 6 months at -80°C before assayed. Patients do not need to rest before the blood extraction unless they have performed a high-endurance exercise. For comparative studies, sex and age should be accounted for as these parameters can impact CGRP concentrations.

**Graphical Abstract:**

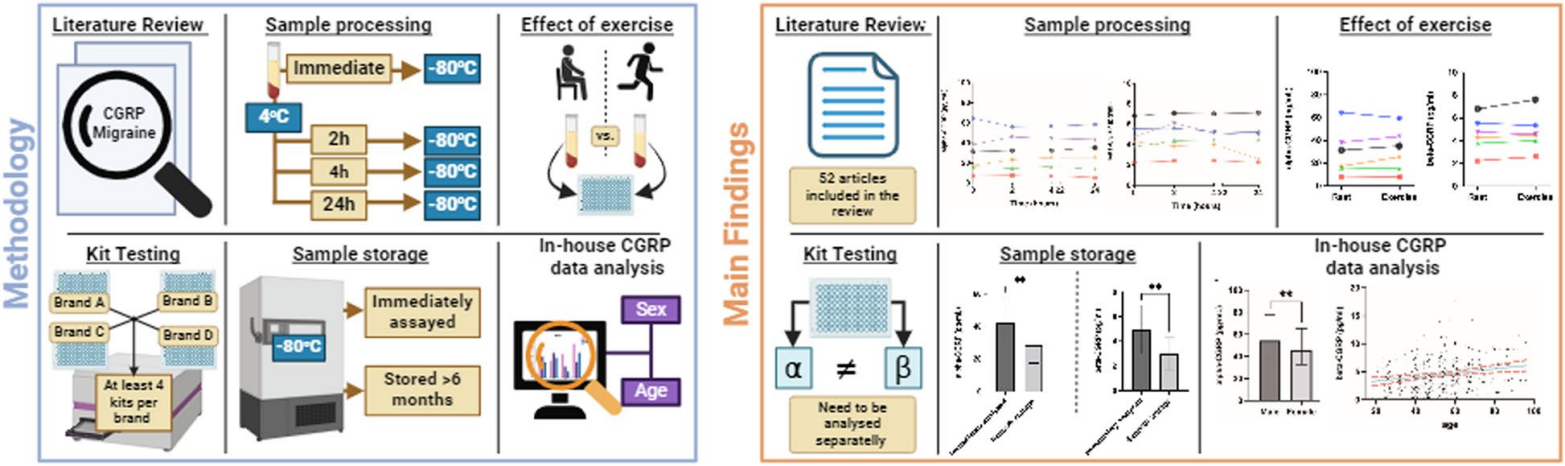

## Introduction

Migraine and its subtypes are diagnosed based on clinical criteria [1]. Thus, multiple phenotypes sharing the same diagnosis are treated the same way with clashing outcomes. However, as many real-world data studies have shown [2], the different phenotypes have been proved ineffective to create profiles prone to respond to the different treatment options. Historical therapies for migraine, which is worth to mention that none of these were initially developed to treat this condition, apart from the triptans, and are not specific for it [3], have not met the challenge of effectively aborting and/or preventing the symptoms, in some cases with limited efficacy, tolerability and patient adherence [4].

Since the 1990s decade our understanding of migraine has expanded markedly and new therapeutic agents have been brought to the market in an effort to alleviate the personal and economic burden that migraineurs suffer. These are the calcitonin gene-related peptide (CGRP)-targeted therapies which have revolutionized the management of migraine [5], including monoclonal antibodies against the CGRP ligand or its receptor [6], and small molecules antagonists to the CGRP receptor, the gepants [7]. Nonetheless, there is still a portion of patients who do not respond to the treatments, highlighting the importance that a biomarker would have in migraine, allowing to create objective diagnostic criteria besides the clinical ones, which may be subject to errors [8], and to monitor objectively the response to treatments.

CGRP is a multifunctional neuropeptide which was first discovered in 1982, described as the result of the alternative splicing of the calcitonin gene (CALCA in humans) transcript, hence its name [9]. Later on, this first form of CGRP will be named alpha-CGRP, as opposed to the beta-CGRP, encoded in a different gene (CALCB in humans), with a different regulation and expression pattern to the alpha-CGRP [10]. These two peptides differ in 3 out of the 37 amino acids of their sequence but share a common structure and are part of the CGRP peptide family, also comprised by calcitonin, adrenomedullin 1 and adrenomedullin 2 [11]. Although their distribution in the human body tends to overlap [12], alpha-CGRP has been described to be the predominant form in the central and peripheral nervous system while beta-CGRP is more relatively abundant in the enteric nervous system [13].

The relevance of the peptide goes beyond its use as a therapeutical target, having been proposed as a biomarker in migraine. Several studies have reported elevation of the peptide in ictal and/or interictal phases in medication-free periods of migraine patients [14–38], the reduction of the CGRP levels after abortive and prophylactic treatment [26, 28, 38–44] and the induction of migraine-like headaches when infused in humans [45]. Despite these results, there are other works contradicting the findings [35, 46–54] and which emphasize the way until its eventual validation and clinical use is still far way to become a reality. The source of such discrepancies, although still unknown, is most probably multifactorial. There is a methodological component [55, 56] and the influence of other individual parameters such as comorbidities [36], concomitant treatments [57] or menstrual cycle [58, 59], which have not been taken into account or which have not been sufficiently described to be considered properly.

In this work we have analysed in detail the existing literature about CGRP measurements in migraine patients, discussing their methodological differences and their effect on the reported concentrations of the peptide. In addition, we have conducted a series of experiments aimed to elucidate the potential effects on serum content of total CGRP, alpha-CGRP, and beta-CGRP, of a number of variables, including different enzyme-linked immunosorbent assay (ELISA) kits, sample processing time, long-term storage or immediate practise of exercise before sampling. Finally, we have analysed our in-house database of CGRP measurements to investigate the effect that sex and age might have on the molecules.

## Methods

### Review of previously published works including CGRP measurements in migraine patients

A systematic search was conducted in the databases PubMed, Scopus and Science Direct until February 2024 using the following terms: (a) CGRP; (b) migraine; and one of the following terms: (c) levels; (d) concentration; (e) measurements. We included original articles with CGRP measurements in humans with migraine. We only included and analysed works written in English language.

### Methodological experiments

#### Kit analysis

We tested 4 different ELISA kit references with serum samples, 2 based on competitive ELISA (Biorbyt, UK, ref: orb438605; BMA Biomedicals, Switzerland, ref: S-1198), specifically designed for the detection of total-CGRP, and 2 based on ELISA sandwich (Abbexa, UK, ref: abx257902; CUSABIO, China, ref: CSB-E08210h), designed for the detection of alpha and beta-CGRP, respectively. All 4 of these products were assayed multiple times (at least 4 for each kit reference) to analyse the optimal dilutions of the samples, their reproducibility and their reported concentrations. All the procedures were carried out strictly following the manufacturer’s instructions of use of their products, they were performed by the same researcher, using the same equipment, and in the same facilities. Regarding the last step of the ELISA processes, in which manufacturers give a window of time, specifying that the user must determine the optimum, we incubated the substrate for 15 min for alpha-CGRP and for 20 min for beta-CGRP. All the samples were measured in duplicate, obtained from morning blood extractions, 9–12 am, from patients in a fast of at least 12h. These samples were let to clot for 10–15 min, centrifuged at 3500 rpm for 10 min and then immediately stored at -80°C until assayed. A standard curve was generated for every single batch, and they were calculated using a 4-parameter logistic (4-PL) regression with r^2^ > 0.999.

#### Influence of sample processing time

We recruited 6 individuals without history of migraine and subjective absence of headache at the day of the sampling (50% male; age range: 24–65 years). These individuals had a blood extraction in the early morning, between 9 am and 9:30 am, performed in rest at our laboratory facilities. The blood was then let to clot for 10 min at room temperature, then centrifuged at 3500 rpm for 10 min to obtain serum. Serum was divided into 4 tubes. First one was immediately stored at -80°C, the other three were kept in the refrigerator at 4°C for, 2, 4 and 24 h respectively before frozen. None of the samples were added peptidase inhibitor. These samples were measured by triplicate.

#### Effect of exercise

Additionally, after the first blood extraction, these same 6 subjects were asked to perform a 20 min run at moderate pace before a second blood extraction. Blood obtained was then processed following the same procedure as the resting samples but in this case all the serum was immediately stored at -80°C. These samples were measured by triplicate.

#### Long-term storage

We assayed 11 consecutive samples from previous works (36.4% male; age range: 26–65) that had been stored at -80°C for more than 6 months and which had been assayed altogether before being stored for a month and before reaching this time point.

### Analysis of our CGRP database

Samples coming from our bio-bank were grouped together, reaching 399 individuals (29.3% male; age range: 18–96 years), and then analysed to see the average levels of the peptide and possible effects of sex and age in the circulating concentrations of the molecules.

### Statistical analysis

Data are displayed as average with standard deviation (SD) unless stated differently. Comparisons between samples immediately processed and stored at -80°C obtained in resting subjects and right after exercising, and samples analysed before and after they had been stored for 6 months were made using the Wilcoxon matched-pairs signed rank test. Comparisons between samples from same individuals that were frozen at different timepoints have been made using Friedman test followed by Dunn’s test. Correlation relationships of the meta-analysis were evaluated by Spearman correlation test and summarized by Spearman’s rho coefficient and related p-values. Comparisons between sub-groups in the meta-analysis were performed using the Mann Whitney U test.

## Results

### Article review

Applying the criteria specified in the method section we included 52 articles from the initial search that have been sorted by sample source and detection methodology and are displayed in Table 1.

**Table 1:** Charasteristics of the studies measuring CGRP in migraine patients

Reference	Disease	Aim of study	Patients	Ictal / Interictal	Patients Sex and Age	Controls	Controls Sex and Age	Sample(s)	Method	Brand	Concentrations	Significant Increase [CGRP] vs HC	Main findings relate to CGRP
[18]	Chronic daily headache (CDH)	Investigating CGRP leveles in the CSF of patients afected by chronic daily headache and halthy controls	20 CDH, 20 Healthy Controls (HC)	Not specified	75% female, 43.6 ± 7.9	Drug-free for at least 2 months and no personal or family history of migraine nor tension-type headache	65% female;, 42.1 ± 8.1	CSF	RIA	Not specified	[Mean ± SD] **Chronic Daily Headache:** 55.23 ± 7.37 pg/mL **HC:** 11.35 ± 2.58 pg/mL	Chronich Daily Headache yes	Increased levels of CGRP in CSF of subjects with chronic dily headache compared to control subjects
[19]	Chornic Daily Hedache	Investigating variations in the levels of glutamate, nitrites and sensory neuropeptides CGRP, substance P and neurokinin A in patients affected by chronic dily headache	25 CDH (15 without analgesic overuse, 10 without anagesic overuse), 20 HC	Not specified	CDH without analgesic overuse: 60% female 43.6 ± 7.9 CDH with analgesic overuse 66.6% female, 45.4 ± 7.2	No medication at the time of sampling, no personal or fmily history of migraine nor sufferred episodic tension-type headache in the last two months	65.5% female, 44.6 ± 8.1	CSF	RIA	Peninsula Laboratories	[Mean ± 2*SEM] **CDH:** 1.26 ± 0.07 fmol/mL C**DH without analgesic overuse:** 1.12 ± 0.06 fmol/mL **CDH with analgesic overuse:** 1.34 ± 0.09 fmol/mL **HC:** 0.78 ± 0.05 fmol/mL	CDH yes CDH with medication overuse yes CDH without medication overuse yes	Increased levels of CGRP in CSF of subjects with chronic dily headache indepently of meeting analgesic overuse criteria compared to control subjects
[22]	Chronic migraine (CM)	Examinating whether the concentrations of endocannabinoids, CGRP and nitrites are altered compared to healthy controls	15 CM, 20 HC	Not specified	73.3% female, 37.4 ± 4.9	Drug-free for at least 2 months and no personal or family history of migraine nor tension-type headache	65% female, 36.3 ± 7.4	CSF	RIA	Peninsula-Laboratories	[Mean ± SEM] **CM:** 44.16 ± 4.63 pmol/L **HC:** 29.37 ± 4.67 pmol/L	CM yes	Increased CGRP concentrations in CSF compared to those measured in controls
[27]	Chronic Migraine	Measuring CGRP levels in the gingival crevicular fluid of individual with CM with aura and comparing the concentrations with those measured in HC	24 CM with aura, 15 HC	Interictal	100% female, 34.83 ± 8.92	Inclusion cirteria not specified	100% female, 35.47 ± 10.74	Gingival Crevicular Fluid	BCA & ELISA	Mybiosource Inc	[Mean ± SD] **CM:** 0.25 ± 0.09 pg/μg **HC:** 0.19 ± 0.07 pg/μg	CM yes	CGRP concentraion in gengival crevicular fluid is increased in chronic migraine compared to HC
[23]	Chronic Migraine	Investigating the relationship between pain intensity and CGRP levels in plasma and saliva	33CM, 36 HC	Not specified	63.6% female, 43.7 ± 18.1	No history of orofacial pain (including headache) within last 6 months	52.8% female, 44.3 ± 14.2	Plasma (CV)	EIA	Spi-Bio	[Mean ± SD] **CM:** 253.6 ± 195.2 pg/mL **HC:** 136.2 ± 92.5 pg/mL	Yes	Statistically increased levels of CGRP compared to HC. Correlation between pain intensity and CGRP levels in CM and correlation between CGRP levels in plasma and saliva
[21]	Migraine	Investgating the levels of SP, CGRP and ACE activity in a sample of migraneurs in interictal periods compared to controls and examinating the correlation between substances	95 Migraneurs (41 Migraine with Aura (MA), 54 Migrine Without Aurwa (MWA)), 52 HC	Interictal	Migrane: 81% female, 30.0 ± 10.4 MA: 73.2% female, 28.3 ± 10.1 MWA: 87.0% female, 31.4 ± 10.2	Withoud headache	75% female, 29.2 ± 9.7	Plasma (CV)	ELISA	Spi-bio	[Mean ± SD] **Migraine:** 19.0 ± 9.1 pg/mL **MA:** 18.8 ± 8.8 pg/mL **MWA:** 19.1 ± 9.4 pg/mL **HC:** 13.4 ± 4.4 pg/mL	Migraine yes MA yes MWA yes	Increased levels of CGRP in interictal phases of both migraine with and whithout aura compared to healthy controls and possible interactions between CGRP, SP, and ACE
[30]	Migraine	Analyzing the relation between CGRP and cytokines during attacks to explore the possible mechanism of migraine	47 Migraine (20 MWA, 27 MA), 38 HC	Ictal	55.3% female, 35.2 ± 9.3	No personal or family history of migraine	57.9% female, 32.4 ± 6.1	Plasma (CV)	ELISA	USCN Life Sciences	[Mean ± SEM] **Migraine:** 80.5 ± 22.3 pg/mL **Migraine without aura:** 74.7 ± 21.6 pg/mL **Migraine with aura:** 84.8 ± 22.2 **HC:** 29.5 ± 8.8 pg/mL	Migraine yes	Levels of CGRP correlated with IL-1B and IL-6 in migraine and elevated levels compared to HC
[31]	Chronic Migraine & Episodic Migraine (EM)	Testing whether CGRP levels in tear fluid are altered in EM and/or CM compared to HC	48 EM, 45 CM, 48 HC	Both	85% female, 36.1 ± 12.1 EM: 88% female, 37.7 ± 12.0 CM: 82% female, 34.1 ± 12.1	Fewer than two mild headache days/month withoutany migraine characteristics	69% female, 33.2 ± 9.6	Plasma (CV)	ELISA	Cusabio	[Mean ± SD] **Interictal Migraine (*n* = 49):** 6.32 ± 3.08 pg/mL **Interical CM (*n* = 19):** 6.24 ± 3.59 pg/mL **Interictal EM (*n* = 30)**: 6.38 ± 2.78 pg/mL **HC:** 6.57 ± 4.25 pg/mL	Interictal Migraine no Interictal CM no Interictal EM no	Plasma CGRP is not increased in interictal migraine patitents
[33]	Chronic Migraine & Episodic Migraine	Comparing interictal concentrations of CGRP and amylin in peripheral bloood between controls and patients with migraine and evaluating their performance in diagnosing CM	191 CM; 58 EM; 68HC	Interictal	CM: 95.3% female, 46.03 ± 11.93 EM: 87.9% female, 37.71 ± 10.47	No personal or famliar history of migraine	86.8% female; 43.58 ± 11.08	Plasma (CV)	ELISA	Bertin Bioreagent	[Mean ± SD] **CM**: 20.01 ± 53.23 pg/mL **EM**: 19.89 ± 26.4 pg/mL **HC:** 11.37 ± 8.3 pg/mL	CM yes EM no	CGRP levels increased in CM compared HC
[35]	Episodic Migraine	Assessing salivary levels of CGRP during migraine attacks and comparing interictal levels in patients with episodic migraine and controls	22 EM, 22HC	Interictal	EM: 100% female; 30.4 ± 9.4	No personal or famliar history of migraine or headache (excluding tension type headache)	100% female; 31.2 ± 11.1	Plasma (CV)	ELISA	Cusabio	[Median (95%CI)] **EM**: 6.0 (4.5–8.4) pg/mL **HC**: 5.1 (3.2–7.1)pg/mL	EM no	Plasma CGRP levels in interictal phases of EM patients are not different from those in HC
[37]	Migraine	Investigating CGRP and PACAP-38 plasma levels in children with migraine and testing their diagnostic value	76 Pediatric migraine (43 ictal; 33 interictal); 77 HC	Both	Migraine group: 54% female; 10.38 ± 3.73	Neurological examination to discard migraine diagnosis	39% female; 9.46 ± 3.91	Plasma (CV)	ELISA	Jiangsu Meimian	[Mean ± SD] **Migraine**: 105.75 ± 13.01 pg/mL **Ictal Migraine (*n* = 43)**: 108.19 ± 9.4 pg/mL **Interictal Migraine (*n* = 33)**: 102.56 ± 16.19 pg/mL **HC**: 85.48 ± 14.58 pg/mL	Migraine yes Ictal yes Interictal yes	Increased CGRP levels during both the ictal and interictl phases of migraine patients compared to HC and the combined use of CGRP and PACAP-38 might be useful to diagnose migraine in children
[50]	Migraine	Exploring the diagnostic accuracy of CGRP and ApoE in migraine	14 Migraine, 14 HC	Both	100% female, 20.29 ± 1.68	No personal or family history of migraine or subjective headache of any type	100% female, 21.43 ± 1.83	Plasma (CV)	ELISA	Biomatik	[Mean ± SD] **Migraine Ictal:** 3.69 ± 2.14 pg/mL **Migraine Interictal:** 3.19 ± 3.36 pg/mL **HC:** 2.02 ± 1.07 pg/mL	Migraine ictal no Migraine interictal no	Serum CGRP levels have a fair diagnostic accuracy to discriinate between migraine ictal phase and HC
[51]	Migraine	Comparing plasma levels of vasoactive petides in pediatric migraine patients without aura with those of age-matched healthy controls	38 MWA (16 ictal, 22 interictal); 20 HC	Both	73.7% female; 13.0 ± 3.3	Without headache	65% female; 11.3 ± 2.9	Plasma (CV)	ELISA	BT Lab	[Mean ± SD] **Migraine:** 213 ± 59 pg/mL **Ictal:** 200 ± 59 pg/mL **Interictal:** 224 ± 59 pg/mL **HC:** 205 ± 41 pg/mL	Migraine no Interictal no Ictal no	CGRP concentrations remained unchanged in both ictal and interictal periods compared to healthy controls
[57]	Chronic Migraine & Episodic Migraine	Identifying possible peripheral biomarkers associated with the two forms of migraine, and with the pressence of medication overuse	27 EM, 28 CM-Medication overuse (MO)	Interictal	EM: 92.6% female, 39.2 ± 8.8 CM: 85.7% female, 47.6 ± 10.9	None	NA	Plasma (CV)	ELISA	Antibodies Online	[Mean ± SD] **EM:** 220.4 ± 83.42 pg/mL **CM-MO before detoxification:** 393.3 ± 242.9 pg/mL **CM-MO responders to detoxification before detoxification:** 322.9 ± 164.1 pg/mL **CM-MO non-responders to detoxification before detoxification:** 717.1 ± 301.1 pg/mL **CM-MO after detoxification:** 275.2 ± 251.8 pg/mL **CM-MO responders to detoxification after detoxification:** 251.8 ± 383.2 pg/mL **CM-MO non-responders to detoxification after detoxification:** 383.2 ± 208.7 pg/mL	NA	CGRP levels are influenced by medication overuse, and modulated by detoxification in subjects with CM-MO
[58]	Episodic Migraine	Assesing CGRP levels at different times of the menstrual cycle	53 EM (25 migraine and endometriosis (EM + E); 28 migraine (EM-E)) 30 HC	Interictal	100% female; EM + E: 35.70 ± 1.32 EM-E: 32.74 ± 1.31	female without any primary headache apart from tensio-type headache and without strong pelvic pain or cramps during menstruation	100% female; 31.55 ± 1.71	Plasma (CV)	ELISA	Bertin Bioreagent	[Median (IQR)] **Perimenstrual:** **EM + E:** 52.59 (35.08–72.41) pg/mL **EM-E:** 47.70 (33.67–73.31)pg/mL **HC:** 55.01 (42.78–130.08)pg/mL **Periovulatory:** **EM + E:** 46.21 (34.10–59.56) pg/mL **EM-E:** 47.39 (34.45–104.33) pg/mL **HC:** 67.34 (49.60–134.06) pg/mL	EM + E no EM-E no	CGRP levels change during the menstrual cycle
[59]	Episodic Migraine	Studying CGRP concentrations in plasma and tear fluid in episodic migraine female patients with regular mentrual cycle (RMC), combined contraception (COC), and in postmenopause (PM)	90 EM (30 EM-RMC; 30 EM-COC; 30 EM-PM), 90 HC (30HC-RMC; 30HC-COC;30HC-PM)	Interictal	100% female; M-RMC: 26.5 (24–30) M-COC: 25 (22.75–30) M-PM: 57.5 (55.75–60)	Without migraine	100% female; HC-RMC: 26 (24–31) HC-COC: 27 (22.75–31) HC-PM: 58.5 (55.75–61.25)	Plasma (CV)	ELISA	Cusabio	[Median (IQR)] **Regular menstrual cycle** **Menstrual EM:** 5.95 (4.37–10.44) pg/mL **Periovulatory EM:** 6.28 (3.56–9.48) pg/mL **Menstrual HC:** 4.61 (2.83–6.92) pg/mL **Periovular HC:** 4.87 (2.95–6.41) pg/mL **Combined contraconcepcion** **Hormone-free interval EM:** 4.87 (4.22–6.15) pg/mL **Hormone intake EM:** 4.92 (3.89–6.24) pg/mL **Hormone-free interval HC:** 6.67 (3.76–8.56) pg/mL **Hormone intake HC:** 6.03 (4.40–9.42) pg/mL **Postmenopause** **EM:** 5.24 (3.89–7.14) pg/mL **HC:** 6.70 (5.48–9.02) pg/mL	Menstrual yes Periovular no Hormone-free interval no Hormone intake no Postmenopause no	Data suggests hormone dependent changes in CGRP concentrations female patients with EM
[60]	Episodic Migraine	Employing a standarized protocol to assess the evolution of CGRP plasma levels during induced migraine attacks under hypoxic challenge	30 EM	Both	EM: 73.3% female; 27.56 ± 7.54	None	NA	Plasma (CV)	ELISA	Bertin Bioreagent	[Mean ± SD] **T0:** 185.19 ± 380.01 pg/mL **T1:** 190.29 ± 391.33 pg/mL **T2:** 192.43 ± 390.31 pg/mL **T3:** 179.81 ± 390.22 pg/mL **T4:** 183.67 ± 383.77 pg/mL **T5:** 179.75 ± 388.92 pg/mL **Toff:** 206.84 ± 400.88 pg/mL **Toff-1:** 212.93 ± 395.67 pg/mL **Toff-2**:184.21 ± 390.78 pg/mL	NA	Plasma CGRP are highly variable in migraine patients and increase during hypoxic challenge
[61]	Chronic Migraine & Episodic Migraine	Assessing the differences of CGRP plasma concentrations during and after cessation of anti-CGRP mAbs	59 including EM and CM at last injection (V1) and 4 months after treatment cesation (V2) 25 Erenumab; 25 Galcanezumab; 9 fremanezumab; 30 Controls	Both (Controls only Interictal)	Erenumab: 92% female, 52(42.5–57.5); Galcanezumab: 96% female, 51.0 (39.5–57.5); Fremanezumab: 100% female, 54.0 (50.5–58.5)	Sex and age paired EM and CM	97% female; 52.0 (45.3–56.3)	Plasma (CV)	ELISA	Bertin Bioreagent	[Median (IQR)] **E(V1)**: 31.2 (25.8–45.6)pg/mL **E(V2):** 30.3 (22.9–47.6)pg/mL **G(V1):** 5439.3(2412.7–6338.1)pg/mL **G(V2):** 1853.2(1136.5–3297.0)pg/mL **F(V1):**29.4(16.4–61.9)pg/mL **F(V2):** 34.3(19.2–62.0)pg/mL **HC:** 32.6(21.3–44.6)pg/mL	NA	Cession of treatment did not have an impact on the free-circulating CGRP concentraions
[62]	Chronic Migraine and Obesity	Testing whether surgical weight loss is effective in migraine improvement through CGRP level reduction	60 CM patients with obesity	Interictal	100% female, 34.83 ± 9.24	None	NA	Plasma (CV)	ELISA	Hangzhou Eastbiopharm	[Mean ± SD] **Basal:** 252.7 ± 56.4 pg/mL **After surgery (6–10 months)**: 130.1 ± 70.5 pg/mL	NA	Bariatric surgery descreases CGRP plasma kevels along with the frequency of migraine attacks
[53]	Cerebral autosomal dominant arteriopathy with subcortical infarcts and leukoencephalopathy (CADASIL) with and without migraine	Determining wether serum CGRP levels differ between CADASIL patients depending on the presence of migraine	28 CADASIL: 18 with migraine 10 without migraine	Interictal	Entire group: 39.3% female, 53.2 ± 14.2 Migraine: 38.9% female, 47.6 ± 13.3 No Migraine: 40% female; 63.3 ± 9.8	None	NA	Plasma (CV)	Peptide extraction + RIA	Phoenix Pharmaceuticals	[Mean ± SD] **Interictal Migraine**: 27.0 ± 9.6 pg/mL **No migraine**: 26.8 ± 15.7 pg/mL	NA	Serum interictal CGRP levels do not differ by the presence of migraine comorbidity in CADASIL patients
[15]	Migraine	Comparing plasma CGRP levels in juvenile migraine patients and in healthy controls	75 EM (45 MWA, 30 MA), 30HC	Both	EM: 42.66% female, 15.94 ± 2.51 MWA: 44.44% female, 16.3 ± 2.6 MA: 40% female, 15.4 ± 2.3	Not specified	50% female, 15.1 ± 2.1	Plasma (CV)	RIA	Peninsula Laboratories	[Mean ± SEM] **Interictal MA:** 34.7 ± 7.2 pmol/L **Interictal MWA:** 39.3 ± 8.6 pmil/L **Ictal MA:** 51.4 ± 7.8 pmol/L **Ictal MWA:** 50.3 ± 6.7 pmol/L **HC:** 38.2 ± 6.5 pmol/L	Ictal MA yes Ictal MWA yes Interictal EM no	Statistically increased levels of CGRP in peripheral circulation samples of migraine patients in the ictal phase compared to interictal phase and HC
[17]	Episodic Migraine	Comparing plasma CGRP levels in adult migraine patients and in healthy controls	20 EM, 20 HC	Interictal	80% female, 40 ± 9	Never has migraine and less than 12 days pr year of tension-type headache	60% female, 41 ± 14	Plasma (CV)	RIA	Schifter S. Circulating concentrations of calcitonin gene-related peptide(CGRP) in normal man determined with a new, highly sensitive radio-immunoassay. Peptides 1991;12:365–369	[Mean ± SEM] **EM:** 75 ± 8 pmol/L **HC:** 49 ± 3 pmol/L	Yes	Statistically elevated levels of CGRP in the peripheral circulation of migarine patients within interictal phase compared to healthy controls
[40]	Migraine	Evaluating the effects of sumatriptan on plasma CGRP concentrion with relation to the drug's animigraine effect	19 MWA	Ictal	100% female, 45 ± 1.4	None	NA	Plasma (CV)	RIA	Nemeth J, Gorcs T, Helyes Zs, Oroszi G, Kocsy T, Pinter E, Szolcsanyi J.Development of a new sensitive CGRP radioimmunoassay forneuropharmacological research. Neurobiology 1998;6:473 – 5	[Mean ± SEM] **Headache responders before sumatriptan (*n* = 6): 16.9** ± 2.8 pmol/L **1-h after sumatriptan reponders (*n* = 6):** 14.7 ± 2.2 pmol/L **Headache non-reponders (*n* = 13): 24.3** ± 2.5 pmol/L **1-h after sumatriptan non-reponders (*n* = 13):** 23.8 ± 2.4 pmol/L	NA	Plasma CGRP concentrations decreases parallel to headache intensity during antimigraine drug therapy and predicts effectiveness of treatment
[48]	Episodic Migraine	Validating the increase of CGRP concentration in jugular blood during migraine attacks	21 EM MWA	Both	80.95% female, 39 (26–53)	None	NA	Plasma (CV)	RIA	Method 1: Goadsby PJ, Edvinsson L, Ekman R. Vasoactive peptide release in the extracerebral circulation of humans during migraine head- ache. Ann Neurol 1990;28: 183–187 Method 2: Peninsula Laboratories	[Mean] **RIA 1** **Ictal:** 16.86 pmol/L **Interictal:** 17.57 pmol/L **RIA 2** **Ictal:** 33.37 pmol/L **Interictal:** 31.84 pmol/L	NA	CGRP levels are not increased in external jugular blood or peripheral blood during migraine withou aura attacks
[63]	Episodic Migraine	Evaluating how plasma CGRP concentration and platelet serotonin content change in peripheral blood circulation during nitroglyceri-induced headache	15 EM WA, 8HC	Both	100% female, 41.9 ± 2.3	Only rare (less than 1 per year) and mild headaches	100% female, 38.5 ± 4.4	Plasma (CV)	RIA	Nemeth J, Gorcs T, Helyes Zs, Oroszi G, Kocsy T, Pinter E, Szolcsanyi J.Development of a new sensitive CGRP radioimmunoassay forneuropharmacological research. Neurobiology 1998;6:473 – 5	[Mean ± SEM] **Migaraneurs who experienced headache (*n* = 10):** **Basal:** 20.2 ± 1.9 pmol/L **Migaraneurs who did not experienced headache (*n* = 5):** **Basal:** 14.0 ± 1.3 pmol/L **Migraneurs:** **Basal:** 18.4 ± 1.7 pmol/L **T1:** 19.7 ± 1.9 pmol/L **T2:** 22.2 ± 2.6 pmol/L **T3:** 21.0 ± 2.4 pmol/L **Controls:** **Basal:** 15.1 ± 2.0 pmol/L **T1:** 17.5 ± 2.3 pmol/L **T2:** 16.5 ± 2.3 pmol/L **T3:** 15.3 ± 2.0 pmol/L	Basal Migrain no	Higher Basal CGRP concentrations are a risk factor to devolp both spontaneous and NO-induces migraine attack
[64]	Episodic Migraine	Testing whether 2 h infusion of VIP can cause alterations in plasma levels of CGRP	19 EM without Aura, 12 HC	Interictal	Not specified	No personal or famliar history of migraine or headache (excluding tension type headache)	Not specified	Plasma (CV)	RIA	Schifter S. Circulating concentrations of calcitonin gene-related peptide(CGRP) in normal man determined with a new, highly sensitive radio-immunoassay. Peptides 1991;12:365–369	[Mean ± SD] **Placebo HC** **T0:** 81.2 ± 15.1 pg/mL **T30:** 78.4 ± 17.4 pg/mL **T60:** 83.3 ± 16.1 pg/mL **T90:** 79.0 ± 20.2 pg/mL **T120:** 85.8 ± 14.6 pg/mL **T150:** 89.5 ± 16.4 pg/mL **T180:** 83.4 ± 16.1 pg/mL **VIP infusion HC** **T0:** 87.9 ± 21.3 pg/mL **T30:** 102.1 ± 27.2 pg/mL **T60:** 109.3 ± 33.3 pg/mL **T90:** 104.1 ± 26.1 pg/mL **T120**: 110.1 ± 26.1 pg/mL **T150**: 97.8 ± 20.7 pg/mL **T180:** 98.2 ± 27.8 pg/mL **Placebo Migraine** **T0:** 68.4 ± 24.3 pg/mL **T30:** 70.6 ± 25.9 pg/mL **T60:** 73.3 ± 23.0 pg/mL **T90:** 73.6 ± 27.2 pg/mL **T120:** 73.9 ± 24.5 pg/mL **T150:** 69.7 ± 24.8 pg/mL **T180:** 71.6 ± 23.7 pg/mL **VIP infusion Migraine** **T0:** 86.9 ± 24.1 pg/mL **T30:** 94.2 ± 25.4 pg/mL **T60:** 95.1 ± 25.2 pg/mL **T90:** 87.5 ± 26.5 pg/mL **T120:** 88.7 ± 26.9 pg/mL **T150:** 85.1 ± 22.4 pg/mL **T180**: 83.3 ± 27.6 pg/mL	Baseline migraine no	Plasma CGRP was elevated in patients with migraine during a prolonged infusion of VIP, but these alterations were not associated with VIP-induced migraine attacks
[65]	Migraine	Investigating the effects od PACAP38 on measured biochemical variables in migraine patients compared to controls who received placebo infusion	32 MWA, 6HC	Both	81,25% female, 48 (25–60)	Inclusion ciriteria not specified	66% female, 42 (24–54)	Plasma (CV)	RIA	Schifter S. Circulating concentrations of calcitonin gene-related peptide(CGRP) in normal man determined with a new, highly sensitive radio-immunoassay. Peptides 1991;12:365–369	Exact values not displayed	Migraine no	PACAP38 infusion do not causes changes in CGRP concentrations
[14]	Migraine	Analyzing the levels of several neuropeptides by local cranial blood sampling of migraneurs during heacache	22 Migraneurs (10 MA, 12 MWA), 12 HC	Ictal	72.72% female, 36 ± 13	Age- and sex-matched	100% female, (19–25)	Plasma (JV)	RIA	Original	[Mean ± SEM] **Migraneurs:** 88.7 ± 5.3 pmol/L **MA:** 92 ± 11 pmol/L **MWA:** 86 ± 4 pmol/L **HC:** < 40 pmol/L	Migraneurs yes MWA yes MA yes	Statistically increased craniovascular CGRP levels in migraneus during ictal phase compared to HC
[16]	Migraine	Verifying whether there are changes in NO metabolites, cGMP, prostaglandins, cAMP, CGRP and NKA during spontaneous migraine attacks	5 MWA	Both	60% female,	None	NA	Plasma (JV)	RIA	Peninsula Labs	[Mean ± SD] **Ictal T0:** 56.8 ± 9.5 pmol/L **Ictal T1:** 76.7 ± 11.3 pmol/L **Ictal T2:** 60.4 ± 10.5 pmol/L I**ctal T4:** 50.2 ± 9.6 pmol/L **Ictal T6:** 46.5 ± 7.8 pmol/L **Interictal:** 33.6 ± 6.2 pmol/L	NA	Confirmed the release of CGRP from trigeminovascular system into the internal jugular venous blood in the course of migraine crises
[39]	Migraine	Evaluating the effect of subcutaneous administation of sumatriptan on CGRP levels during acute migraine episodes	8 Migraneurs	Ictal	87.5% female, 34.25 ± 5.62	None	NA	Plasma (JV)	RIA	Goadsby PJ, Edvinsson L, Ekman R. Vasoactive peptide release in the extracerebral circulation of humans during migraine head- ache. Ann Neurol 1990;28: 183–187	[Mean ± SE] **Pre-treatment:** 60 ± 8 pmol/L **Post-treatment**: 40 ± 8 pmol/L	NA	Statistically decrease of CGRP levels after sumatriptan administration as an abortive treatment of migraine ictal phase
[41]	Migraine without aura	Investigating the clinical and biochemical correltion between satisfactory and poor response to rizatriptan	20 MWA	Both	Not specified	None	NA	Plasma (JV)	RIA	Peninsula Laboratories	[Mean ± SEM] **Responders:** **Before rizatriptan administration:** 12.2 ± 3.2 pmol/L **1 h:** 7.6 ± 2.1 pmol/L **2 h:** 3.4 ± 2.1 pmol/L **4 h:** 2.6 ± 1.1 pmol/L **6 h**: 2.3 ± 0.9 pmol/L **12 h:** 2.1 ± 0.8 pmol/L **Non-responders** **Before rizatriptan administration:** 7.4 ± 2.4 pmol/L **1 h:** 7.6 ± 2.1 pmol/L **2 h:** 7.9 ± 3.1 pmol/L **4 h**: 7.7 ± 3.3 pmol/L **6 h:** 7.3 ± 2.9 pmol/L **12 h:** 7.2 ± 3.1 pmol/L	NA	Response to rimatriptan is associated with higher levels of trigemnial activation biomarker, CGRP
[47]	Migraine	Examinating whether release of CGRP and others vasoactive neuropeptides takes place during migraine attacks in the jugular vein	8 Migraneurs	Both	62.5% female, 40.4 (30–53)	None	NA	Plasma (JV)	RIA	Goadsby PJ, Edvinsson L, Ekman R. Vasoactive peptide release in the extracerebral circulation of humans during migraine head- ache. Ann Neurol 1990;28: 183–187	[Mean] **Ictal migraneurs (*n* = 4)**: 65 pmol/L **Interictal migraneurs (*n* = 4)**: 68 pmol/L	NA	No differences in CGRP levels between induced migraine attacks and non induced in migraine patients
[48]	Episodic Migraine	Validating the increase of CGRP concentration in jugular blood during migraine attacks	17 EM MWA	Both	80.95% female, 39 (26–53)	None	NA	Plasma (JV)	RIA	Method 1: Goadsby PJ, Edvinsson L, Ekman R. Vasoactive peptide release in the extracerebral circulation of humans during migraine head- ache. Ann Neurol 1990;28: 183–187 Method 2: Peninsula Laboratories	[Mean] **RIA 1** **Ictal:** 17.18 pmol/L **Interictal:** 15.88 pmol/L **RIA 2** **Ictal:** 32.59 pmol/L **Interictal:** 30.59 pmol/L	NA	CGRP levels are not increased in external jugular blood or peripheral blood during migraine without aura attacks
[23]	Chronic Migraine	Investigating the relationship between pain intensity and CGRP levels in plasma and saliva	33CM, 36 HC	Not specified	63.6% female, 43.7 ± 18.1	No history of orofacial pain (including headache) within last 6 months	52.8% female, 44.3 ± 14.2	Saliva	ELISA	Spi-Bio	[Mean ± SD] **CM:** 431.6 ± 272.8 pg/mL **HC:** 301.5 ± 188.9 pg/mL	Yes	Statistically increased levels of CGRP compared to HC. Correlation between pain intensity and CGRP levels in CM and correlation between CGRP levels in plasma and saliva
[35]	Episodic Migraine	Assessing salivary levels of CGRP during migraine attacks and comparing interictal levels in patients with episodic migraine and controls	22 EM, 22HC	Both	EM: 100% female; 30.4 ± 9.4	No personal or famliar history of migraine or headache (excluding tension type headache)	100% female; 31.2 ± 11.1	Saliva	ELISA	Cusabio	[Median (95%CI)] **Interictal EM:** 98.0 (56.6–124.0) pg/mL; **HC**: 54.3 (42.2–70.1)pg/mL [Marginal Mean (95%CI)] **Preictal EM (*n* = 49):** 169.0 (104.2–234.0) pg/mL **Ictal EM (*n* = 49)**: 247.0 (181.9–312.0) pg/mL **After 2 h EM (*n* = 49):** 143.0 (77.7–208.0) pg/mL **After 8 h EM (*n* = 49)**: 169.0 (103.5–234.0) pg/mL **Post-ictal EM (*n* = 49)**: 173.0 (107.8–238.0) pg/mL	Interictal EM yes	Salivary CGRP levels vary according to the migraine phase
[36]	Chronic Migraine & Episodic Migraine	Analyzing salivary CGRP levels in migraine patients to predict erenumab response	Basal: 27 EM (7 EM post-treatment), 16 CM, 27 HC	Both	EM: 96.3% female; 35.0 (24.5–40). CM: 87.5% female; 41.5 (34.8–48.5)	No personal or famliar history of migraine or headache (excluding tension type headache)	96.3% female; 32.0 (21.5–41.0)	Saliva	ELISA	Cusabio	[Mean ± SD] **Basal:** **HC without depression (*n* = 25)**: 75.97 ± 27.46 pg/mL **HC with depression (*n* = 2):** 90.82 ± 108.50 pg/mL **EM without depression (*n* = 24):** 198.90 ± 27.89 pg/mL **EM with depression (*n* = 3)**: 213.67 ± 78.27 pg/mL **CM without depression (*n* = 10):** 194.34 ± 43.75 pg/mL **CM with depression (*n* = 6): 460**.69 ± 57.24 pg/mL **M3:** **EM without depression (*n* = not displayed):** 235.05 ± 59.32 pg/mL **EM with depression (*n* = not displayed):** not displayed **CM without depression (*n* = 10):** 256.34 ± 47.56 pg/mL **CM with depression (*n* = 6):** 446.99 ± 63.44 pg/mL	EM basal without depression yes EM basal with depression yes CM basal without depression yes EM M3 without depression yes EM M3 with depression no CM M3 without depression yes CM M3 with depression yes	Patients with high frequency episodic and chronic migraine do not have higher CGRP leveles compared to controls and drepression symptoms seems to increase CGRP levels
[46]	Migraine without aura and clusted headache	Measuring VIP, substance-P and CGRP levels in saliva of different primary headache disorders and comparing them with controls	15 Migraine without aura 10 Episodic cluster headache during cluster period 5 Episodic cluster headache out of cluster period 34 Healthy controls	Both	Migraine without aura: 53% female, 43 (31–61) Episodic cluster headache during cluster period: 10% female, 45.8 (29–62) Episodic cluster headache out of cluster period: 20% female, 40.7 (32–56) 34 Healthy controls	No personal nor familiar history for idipathic headache	53% female, 43.7 (26–63)	Saliva	RIA	Maggi CA, Santicioli P, Geppetti P, et al. Simultaneous release of substance P- and calcitonin gene-related peptide (CGRP)-like immunoreactivity from isolated muscle of the guinea pig urinary bladder. Neurosci Lett. 1988;87(1):163–167	[Mean ± SEM] **Ictal migraine:** 27.3 ± 2.9 pmol/L **Interictal migraine:** 14.3 ± 2.5 pmol/L **Healthy controls:** 22.0 ± 1.7 pmol/L **Cluster headache attack:** 53.7 ± 5.2 pmol/L **Cluster headache between attacks:** 40.1 ± 2.3 pmol/L **Cluster headache outside cluster period**: 33.4 ± 7.7 pmol/L	Interictal Migraine no Ictal Migraine no	CGRP increases its concentrarion from basal both during migraine and cluster headache attacks
[20]	Migraine and Rhinosinusitis	Comparing CGRP and VIP saliva levels in subjects experiencing noninfectious allergic rhinosinusitis, migraine with sinus symptoms, and no symptoms	5 Migraneurs with sinus symptoms 5 Allergic rhinosinusitis 5 HC	Both	Not specified	No history of migraine, self-described sinus headache, or symptoms of allergic rhinosinusitis within the previous 6 months	Not specified	Saliva	RIA + Bradford	Peninsula Laboratories—Bachem	[Mean] **Interictal Migraneurs:** 53 pmol/mg of total protein **Ictal Migraneurs:** ~ 65 pmol/mg of total protein **After 2 h of cessation of attackin migraneurs:** ~ 25 pmol/mg of total protein **Rhinosinusitis:** 24 pmol/mg of total protein **HC:** ~ 10 pmol/mg of total protein	Interictal Migraine yes Ictal Migraines yes	Correlation between CGRP and VIP levels increase during attacks and elevated concentrations of CGRP in migraneurs compared to HC in saliva
[42]	Episodic Migraine	Measuring CGRP levels in the saliva of individuals with migraine during the premonitory period, mild headache, moderate to severe headache, and post-resolution phases as compared with baseline	22 EM	Both	90.9% female, 38.9 ± 2.7	None	NA	Saliva	RIA + Bradford	Pensinsula Laboratories—Bachem	[Mean ± SEM] **Interictal:** 58.2 ± 1.6 pmol/mg of total protein **Premonitory:** not displayed **Mild Pain:** not displayed **Moderate Pain:** not displayed **Resolution:** not displayed	NA	Increased CGRP levels during moderate pain phase compared to baseline and levels restored at the time of headache resolution
[43]	Chronic Migraine	Testing whether CGRP levels in saliva are altered in CM patients as a consequence of botox treatment	20 CM (10 tretaed with botox; 10 placebo)	Interictal	75% female, 48.5 ± 12.87	CM treated with saline solution (placebo group)	NA	Saliva	RIA + Bradford	Pensinsula Laboratories—Bachem	[Mean ± SEM] **Basal CM (*n* = 20):** 32 ± 3 pmol/mg total protein **Month 1 Botox Group (*n* = 10)**: 39.4 ± 7.5 pmol/mg total protein **Month 3 Botox Group (*n* = 10):** 25.5 ± 4.1 pmol/mg total protein	NA	Onabotulinumtoxin A reduces salivary leveles of CGRP
[24]	Episodic Migraine	Investigating the presence of endothelial dysfunction in patients with migraine during interictal and ictal periods	47 EM (33 MWA; 14 MA), 23 HC	Both	97.89% female; 37.8 ± 10.4	Without migraine or other type of headache	95.65% female, 31.8 ± 11.0	Serum (CV)	ELISA	Peninsula Laboratories	[Mean ± SD] **Interictal EM:** 164.2 ± 139.1 pg/mL **Interictal MWA:** 151.5 ± 140.2 pg/mL **Interictal MA:** 197.1 ± 136.6 pg/mL **Ictal EM (*n* = 19):** 298.2 ± 100.3 pg/mL **HC:** 37.1 ± 38.5 pg/mL	Interictal EM yes Ictal EM yes	Statitically increased levels of CGRP compared to HC in both ictal and interictal phases
[25]	Chronic Migraine, Episodic Migraine & Cluster Headache	Analyzing the potential role of CGRP as biomarker for permanent trigeminovascular activation	103 CM, 43 EM, 14 CH, 31HC	Interictal	CM: 100% female, 43.1 ± 11.7 EM: 100% female, 44.4 ± 11.6 CH: 100% female, 45.4 ± 7.9	No headache history	100% female, 38.6 ± 12.8	Serum (CV)	ELISA	USCN Life Sciences	[Mean ± SD] **CM:** 74.90 ± 28.29 pg/mL **EM:** 46.37 ± 15.21 pg/mL **CH:** 45.87 ± 12.32 pg/mL **HC:** 33.74 ± 16.10 pg/mL	CM yes EM yes CH no	Increased CGRP levell in interictal phases in the absence of symptomatic medication in CM and EM
[26]	Chronic Migraine	Analyzing the potential relationship between interictal CGRP and VIP levels and response to OnabotA treatment response in CM	81 CM, 33HC	Interictal	95.1% female, 46.2 ± 11.0 (23–65)	No headache history	100% female, 39.4 ± 13.2 (21–61)	Serum (CV)	ELISA	USCN Life Sciences	[Mean ± SD] **CM:** 64.9 ± 31.0 pg/mL **CM non-responders:** 48.3 ± 21.2 pg/mL **CM responders:** 70.4 ± 31.9 pg/mL **HC:** 33.3 ± 15.7 pg/mL	CM yes Responders yes Non-responders yes	Interictal levels of CGRP could act as a response biomarker for OnabotA
[28]	Chronic Migraine	Identifying biomarkers in peripheral bloodthat can predict outcome for OnabotA treatment in CM	62 CM (47 responders, 15 non-responders), 24 HC	Interictal	CM: 96.77% female, 42.35 ± 12.46 Responders: 97.9% female, 39.4 ± 12.0 Non-responders: 93.3% female, 51.6 ± 9.1	No headache history nor chonic pain conditions or chronic anti-inflammatory treatment	95.8% female, age not displayed	Serum (CV)	ELISA	Phoenix Pharmaceuticals	[Mean ± SD] **CM:** 115.0 ± 92.9 ng/mL **Responders:** 133.1 ± 86.6 ng/mL **Non-responders:** 58.2 ± 91.7 ng/mL **HC:** 26.9 ± 12.5 ng/mL	CM yes Responders yes Non-responders yes	CGRP levels are associated with response to OnabotA
[29]	Chronic Migarine	Invesrigating the relationship between periodontitis and CGRP in chronic migraine	102 CM, 77 HC	Interictal	98.0% female, 47.0 ± 10.2	Without neurological disorders	97.4% female, 47.5 ± 8.9	Serum (CV)	ELISA	Cloud-Clone Corp	[Mean ± SD] **CM:** 17.9 ± 6.7 pg/mL **HC:** 6.8 ± 4.2 pg/mL	CM yes	Periodontal inflammation is linked with higher serum CGRP levels in patients with chronic migraine
[32]	Chronic Migraine & Episodic Migraine	Comparing CGRP, VIP and PACAP interictal serum levls in a case–control study of CM, EM and HC and assesing their possible diagnostic value	101CM, 98 EM, 97 HC	Interictal	CM: 88.1% female, 41 ± 10 EM:90.8% female, 41 ± 10	No acute headache nor chronic pain	90.7% female, 41 ± 10	Serum (CV)	ELISA	Cloud-Clone Corp	[Median (IQR)] **CM:** 18.023 (14.4–24.7) pg/mL **CM with OnabotA treatment (*n* = 42):** 20.725 (16.56–30.11) pg/mL **CM with preventive treatment (*n* = 27):** 17.569 (14.9–21.9) pg/mL **CM without preventive treatment (*n* = 32):** 13.479 (9.6–19.72) pg/mL **EM:** 14.659 (10.29–17.45) pg/mL **HC:** 13.988 (10.095–17.87) pg/mL	CM yes EM no	CGRP levels increased in CM compared to EM and HC regardless of preventive treatments
[34]	Migraine	Revealing the diagnostic value of CGRP and PTX-3 in acute migraine	85 Migraine, 50HC	Ictal	63.5% female, 28.28 ± 7.45	No chronical medical conditions like migraine nor any history of migraine in the family	62% female, 28.08 ± 6.48	Serum (CV)	ELISA	Bioassay Technology Laboratory	[Median (Range)] **Migraine:** 146.70(21.52–413.67) pg/mL **Migraine with aura (*n* = 31):** 154.71 (49.76–390.94) pg/mL **Migraine without aura (*n* = 54):** 146.70 (21.52–413-67) pg/mL **HC:** 65.90 (3.80–256.60) pg/mL	Migraine yes	CGRP serum levels are higher in migraine attacks than in the control group and can be used as valuable diagnostic biomarkers
[38]	Chronic Migraine	Analyzing the evolution of alpha and beta-CGRP circulating levels though-out CGRP monoclonal amntibodies treatment in patients with chronic migraine	96 CM, 78 HC	Interictal	86.5% female, 50.0 ± 9.9	No personal or famliar history of migraine	73.1% female, 52.9 ± 17.6	Serum (CV)	ELISA	Abbexa (alpha-CGRP) & Cusabio (beta-CGRP)	[Median (95%CI)] **Alpha-CGRP:** **CM M0**: 47.7 (38.9–54.1) pg/mL **CM M0.5**: 40.4 (35.6–48.1) pg/mL **CM M3**: 40.9 (36.3–45.9) pg/mL **HC**: 37.5 (33.9–45.0) pg/mL **Beta-CGRP**: **CM M0**: 4.3 (3.3–5.0) pg/mL **CM M0.5**: 4.5 (3.5–5.2) pg/mL **CM M3**: 4.6 (3.7–5.2) pg/mL **HC**: 4.4 (3.4–5.6) pg/mL	M0 Migraine Alpha CGRP yes M0.5 Migraine Alpha CGRP no M3 Migraine Alpha CGRP no M0 Migraine Beta CGRP no M0.5 Migraine Beta CGRP no M3 Migraine Beta CGRP no	Treatment with anti-CGRP mAb is able to prgressively normalize basally icreased alpha-CGRP levels in CM and its effect correlates with efficacy measurements
[44]	Chronic Migraine	Testing whether treatment with OnabotA is able to induce changes in serum CGRP concentrations	83 CM	Interictal	94% female, 44.0 ± 12.0 (20–65)	None	NA	Serum (CV)	ELISA	USCN Life Sciences	[Median (range)] **Pre-treatment:** 74.09 (11.4–241) pg/mL **Pre-treatment responders (*n* = 64):** 76.85 pg/mL **Pre-treatment non-responders (*n* = 19)**: 50.45 pg/mL **1-month after onobotA treatment:** 51.89 (10.2–199.4) pg/mL **1-month after onobotA treatment responders:** 52.48 pg/mL **1-month after onobotA treatment non-responders:** 51.89 pg/mL	NA	Onabotulinumtoxin A reducecscirculating leveles of CGRP in peripheral blood samples
[49]	Chronic Migraine & Episodic Migraine	Validating the role of interictal serum CGRP concetrations in peripheral blood as a diagnostic biomarker for chronic migraine	99 EM, 44 CM, 27 HC	Both	EM: 78.8% female, 44 (31–49) CM: 81.8% female, 39.5 (31–54)	No subjective headache nor chonic pain conditions or chronic treatment	92.6% female, 34 (27–42)	Serum (CV)	ELISA	USCN Life Sciences	[Mean ± SD] **Interictal CM (*n* = 34):** 64.9 ± 15.32 pg/mL **Interictal EM (*n* = 96):** 67.0 ± 20.70 pg/mL **HC:** 75.7 ± 20.07 pg/mL	Interictal CM no Interictal EM no	Serum interictal CGRP levels are not elevated in CM nor in EM
[54]	Migraine with or witout aura	Comparing CGRP levels between ictal and interictal phases and with healhty controls	30 Migraneurs 25 Healthy controls	Both	87% female, 25 (18–41)	No primary headache disorders, hypertension, renal dysfunction, endocrinological or rheumatological disease nor signs of active infetion	84% female, 25 (22–40)	Serum (CV)	ELISA	Elabscience	[Median (IQR)] **Ictal:** 2.93 (2.45–3.90) pg/mL **Interictal:** 3.25 (2.85–4.67) pg/mL **Control group:** 3.03 (2.48–3.80) pg/mL	Ictal no Interictal no	Interictal and ictal levels of CGRP are similar to controls
[66]	Chronic Migraine	Assesing the effect of ultrasound-guided bilateral greater occipital nerve blocking (GONB) in chronic migraine patients and its relationship to serum CGRP levels	40 CM	Interictal	77.5% female, 31.1 ± 7.3	None	NA	Serum (CV)	ELISA	Norvus Biologicals	[Median (IQR)] **Basal:** 145 (60–380) pg/mL **1-month after GONB**: 40 (25–60) pg/mL	NA	GONB treatment reduces serum CGRP levels
[67]	Chronic Migraine	Analysing the possible correlation between cranial autonomic parasympathetic symptoms (CAPS) and the serum levels of VIP and CGRP	87 CM	Interictal	94.3& female, 44.7 ± 10.6 (19–65)	None	NA	Serum (CV)	ELISA	USCN Life Sciences	[Median (range)] 61.4 (11.4–157.7) pg/mL	NA	Serum CGRP levels did not correlate with presence of CAPS
[52]	Chronic Migraine & Episodic Migraine	Investigating how CGRP levels change before and after treatment with erenumab and evaluating the association with the clinical response	94 Erenumab treated (including EM and CM)	Both	84% female; 42 ± 12.6	None	NA	Serum (CV)	RIA	Phoneix Pharmaceuticals	[Median (IQR)] **Basal**: 14.1 (8.2–33.9) pg/mL; **T1**: 13.8 (7.0–33.1) pg/mL	NA	Lower serum CGRP after starting treatment was associated with a higher rreduction in migraine days after three months of treatment
[68]	Migraine	Investigating the effect of soy isoflavones on migraine characteristics and CGRP levels in female	88 MWA: 44 Placebo 44 Soy isoflavones	Not specified	100% female; Placebo: 35.72 ± 5.61 Soy Isoflavones: 33.77 ± 8.63	None	NA	Serum (Not Specified)	ELISA	Crystal Day Biotec	Exacts values not displayed in text nor tables, only figures	NA	Soy isoflavones significantly reduce CGRP levels
[27]	Chronic Migraine	Measuring CGRP levels in the gingival crevicular fluid of individual with CM with aura and comparing the concentrations with those measured in HC	24 CM with aura, 15 HC	Interictal	100% female, 34.83 ± 8.92	Inclusion cirteria not specified	100% female, 35.47 ± 10.74	Serum (Not Specified)	ELISA	Mybiosource Inc	[Mean ± SD] **CM:** 41 ± 16 pg/mL **HC: 2**9 ± 8 pg/mL	CM yes	CGRP concentraion inserum is increased in chronic migraine compared to HC
[31]	Chronic Migraine & Episodic Migraine	Testing wether CGRP levels are altered in EM and/or CM compared to HC	48 EM, 45 CM, 48 HC	Both	85% female, 36.1 ± 12.1 EM: 88% female, 37.7 ± 12.0 CM: 82% female, 34.1 ± 12.1	Fewer than two mild headache days/month withoutany migraine characteristics	69% female, 33.2 ± 9.6	Tear fluid	ELISA	Cusabio	[Mean ± SD] **Interictal Migraine (*n* = 49)**: 1.10 ± 1.27 ng/mL I**nterictal CM: (*n* = 19)**1.10 ± 1.47 ng/mL **Interictal EM (*n* = 30)**: 1.09 ± 0.89 ng/mL **HC**: 0.75 ± 0.80 ng/mL	Interictal Migraine yes Interictal CM no Interictal EM no	Tear fluid CGRP is significantly increased in interictal migraine patitents
[59]	Episodic Migraine	Studying CGRP concentrations in plasma and tear fluid in episodic migraine female patients with regular mentrual cycle (RMC), combined contracpetion (COC), and in postmenopause (PM)	90 EM (30 EM-RMC; 30 EM-COC; 30 EM-PM), 90 HC (30HC-RMC; 30HC-COC;30HC-PM)	Interictal	100% female; M-RMC: 26.5 (24–30) M-COC: 25 (22.75–30) M-PM: 57.5 (55.75–60)	Without migraine	100% female; HC-RMC: 26 (24–31) HC-COC: 27 (22.75–31) HC-PM: 58.5 (55.75–61.25)	Tear fluid	ELISA	Cusabio	[Median (IQR)] **Regular menstrual cycle** **Menstrual EM**: 1.20 (0.36–2.52) ng/mL **Periovulatory EM**: 0.70 (0.18–2.29) ng/mL **Menstrual HC:** 0.4 (0.14–1.22) ng/mL **Periovular HC:** 0.63 (0.14–1.22) ng/mL **Combined contraconcepcion** **Hormone-free interval EM:** 0.46 (0.10–1.01) ng/mL **Hormone intake EM:** 0.32 (0.09–1.44) ng/mL **Hormone-free interval HC:** 0.36 (0.14–0.59) ng/mL **Hormone intake HC:** 0.40 (0.13–0.82) ng/mL **Postmenopause** **EM:** 0.70 (0.34–1.50) ng/mL **HC:**0.43 (0.21–1.01) ng/mL	Menstrual yes Periovular no Hormone-free interval no Hormone intake no Postmenopause no	Data suggests hormone dependent changes in CGRP concentrations female patients with EM

Out of these 52 articles, the main source of sample were blood extractions, with 44 (84.6%) works performing them. Twenty-eight (53.8%) used plasma samples, 6 (11.5%) from the jugular vein and the remaining 22 (42.3%) from the cubital vein. Serum was employed in 16 (30.8%) of the studies. Continue by order of use, saliva was the third sample source with 7 (13.5%), followed by cerebrospinal fluid (CSF) with 3 (5.8%) and by tear fluid with 2 (3.8%), and last, gingival crevicular fluid (GCF) with 1 (1.9%). According to the determination method, 21 (40.4%) of the studies measured CGRP by radioimmunoassay (RIA), 2 (3.8%) of them together with Bradford protein assay (Bradford), and 29 (55.8%) by ELISA, 1 (1.9%) performed along with bicinchoninic acid protein assay (BCA), and 2 (3.8%) used undefined enzyme immune assay (EIA).

Seventeen (32.7%) studies did not include healthy controls while the remaining 35 (67.3%) did. Sampling of the migraine patients were performed only in the ictal phase for 5 (9.6%) studies, only in the interictal phase for 20 (38.5%), in both phases for 22 (42.3%) works, and in 5 (9.6%) of them the phase was not specified.

Data was presented in different ways including mean ± standard deviation, ± standard error of mean (SEM), ± 2*SEM; median with range, interquartile range (IQR), 95% confidence intervals (CI), and in multiple units, pmol/L, fmol/mL, pmol/mg of total protein, pg/mL, pg/µg of total protein.

Therefore, these methodologies, sampling differences and variable data displays did not allow for a meta-analysis, and the absolute CGRP range among all the studies could be inferred, showing a wide range of concentrations (2.45–219,700 pg/mL) [28, 54].

### Experimental results

#### Kit analysis

The kit from Biorbyt showed an elevated content of CGRP (range: 150-980pg/mL) compared to what has been reported in the bibliography [24–27, 29, 38, 49, 54] when undiluted serum samples were used. Moreover, the reproducibility of the kit was not satisfactory as the assayed samples did not meet the intra and inter-assay coefficient of variance criteria set by the manufacturer (> 10% and > 12%, respectively). This kit also showed a total lack of linearity for the dilutions of 1:2, 1:4, 1:8, 1:16 and 1:32 with each dilution showing higher CGRP concentrations than the one before (data not shown).

For BMA Biomedical kit we were unable to obtain a single measurement within the detection range. Since we decided to strictly follow the manufacturer’s instructions, we could not modify the standard curve points. All the readout absorbance measurements from the tested samples exceed the absorbance range obtained from the readout of the standard curve, and because this is a competitive ELISA technique, no dilution could be tested and neither we could assayed the reproducibility of the test.

Alpha-CGRP specific kit, from Abbexa, showed similar CGRP concentrations (range: 25-105pg/mL) to what has been described previously in most studies using serum from our group [38, 69, 70] and others [25–27, 49]. Most of the samples fall within mid-range of the standard curve but the kit showed a good linearity of the measurements when samples were diluted 1:2, 1:3, 1:4 and 1:8 (data not shown). Across the different plates results fulfilled the reproducibility criteria by having an intra and inter-assay coefficient of variance below the maximum set by the manufacturer (< 8% and < 10%, respectively).

The last kit, from CUSABIO, showed similar beta-CGRP concentrations than reported in the literature (range: 1.6–10.5pg/mL) [31, 35, 36, 38, 70, 71]. Because the samples fall within the lower part of the standard curve dilution of 1:2, 1:3 and 1:4 resulted in a lack of signal and the impossibility to determine the concentration of the peptide in all the samples but those with the higher beta-CGRP content. In this latter group the linearity found was between the ranges supplied by the manufacturer. Across the different plates results fulfilled the reproducibility criteria by having an intra and inter-assay coefficient of variance below the maximum set by the manufacturer (< 8% and < 10%, respectively).

Because the 2 kits based on competitive ELISA did not meet the quality requirements and did not adjust to the reported units in the literature the following experiments were carried out using the kits from Abbexa and CUSABIO which have been used by our group in previous studies [38, 69–71].

#### Influence of sample processing time

**Fig. 1:**
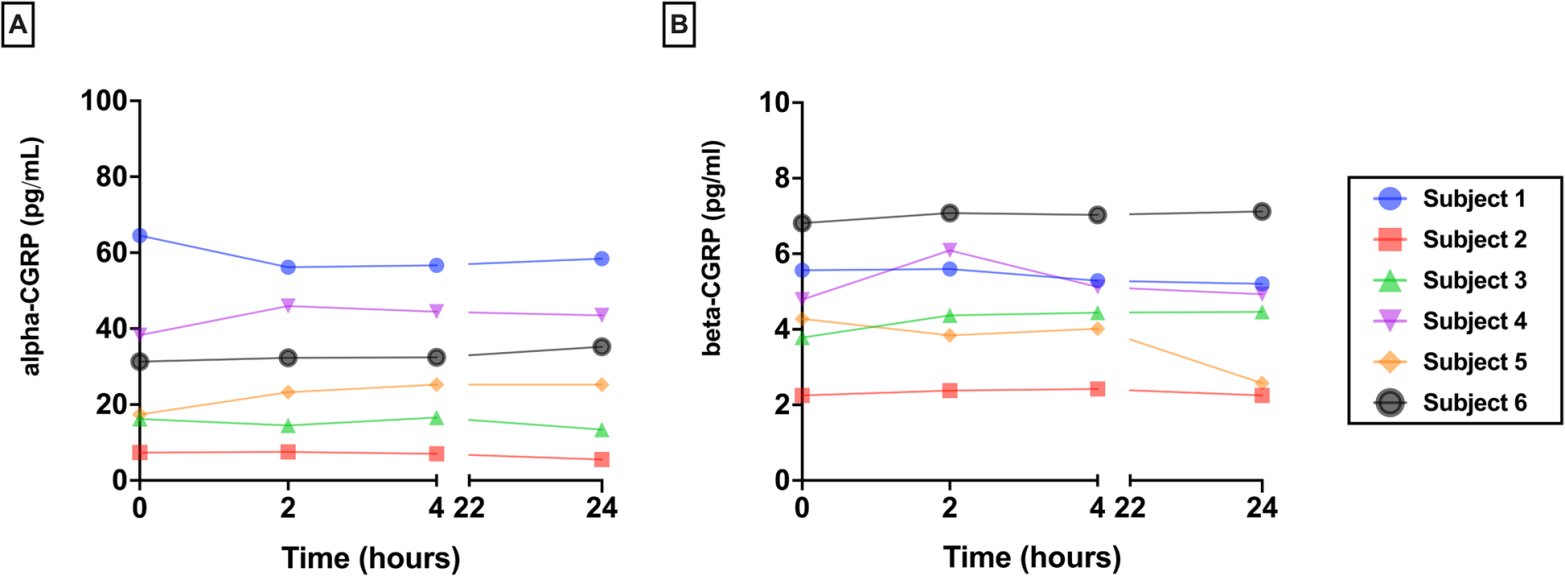
Sample processing: evolution of individual **A** alpha-CGRP and **B** beta-CGRP values for each subject throughout the time samples remained stored at 4°C before froze at -80°C

We did not find changes in alpha nor beta-CGRP across samples which remained for 2 h (alpha: 29.9 ± 18.6pg/mL; beta: 4.9 ± 1.7pg/mL), 4 h (alpha: 30.4 ± 18.2pg/mL; beta: 4.7 ± 1.5pg/mL) and 24 h (alpha: 30.2 ± 19.6pg/mL; beta: 4.4 ± 1.8pg/mL) at 4°C compared to those which got deep frozen right away (alpha: 29.2 ± 20.6pg/mL; beta: 4.6 ± 1.6pg/mL; *p* = 0.99; *p* = 0.84; *p* = 0.99; respectively) (Fig. 1).

#### Effect of exercise

**Fig. 2:**
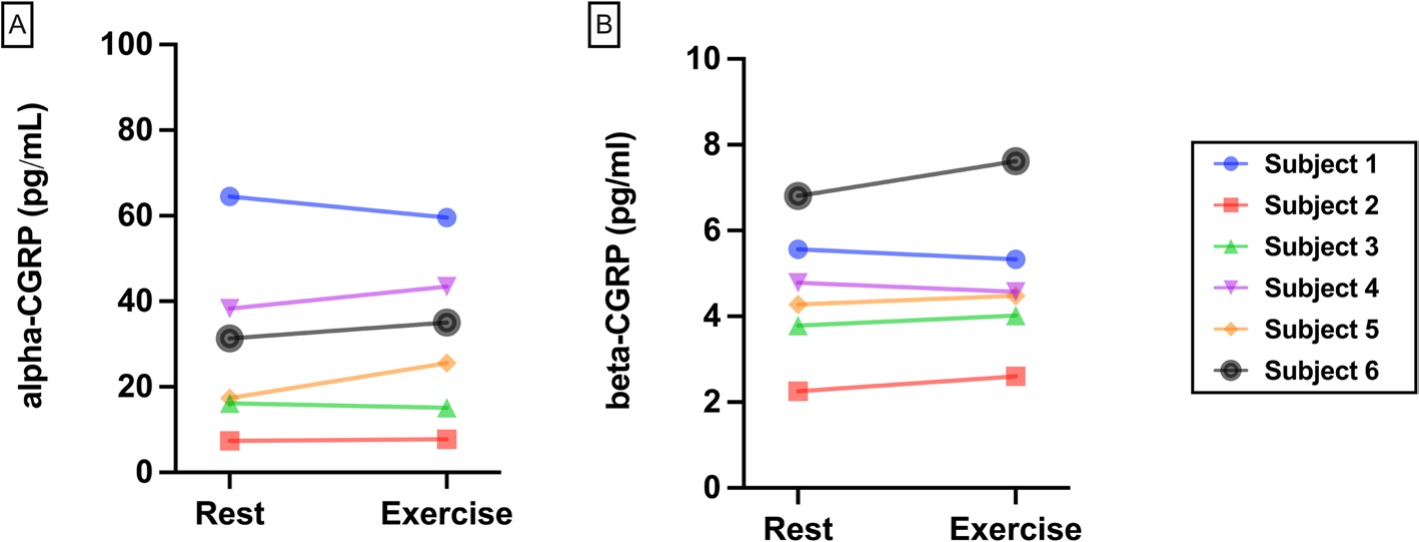
Effect of exercise: evolution of individual **A** alpha-CGRP and **B** beta-CGRP values for each subject when sampling was performed in rest of after 20 minutes of moderate exercise

No differences were found in none of the molecules when comparing serum samples obtained in rest an immediately stored at -80°C and those obtained after exercise and with the same processing protocol (alpha: 31.1 ± 19.0pg/mL; beta: 4.8 ± 1.7pg/mL; *p* = 0.44) (Fig. 2).

#### Long-term storage

**Fig. 3:**
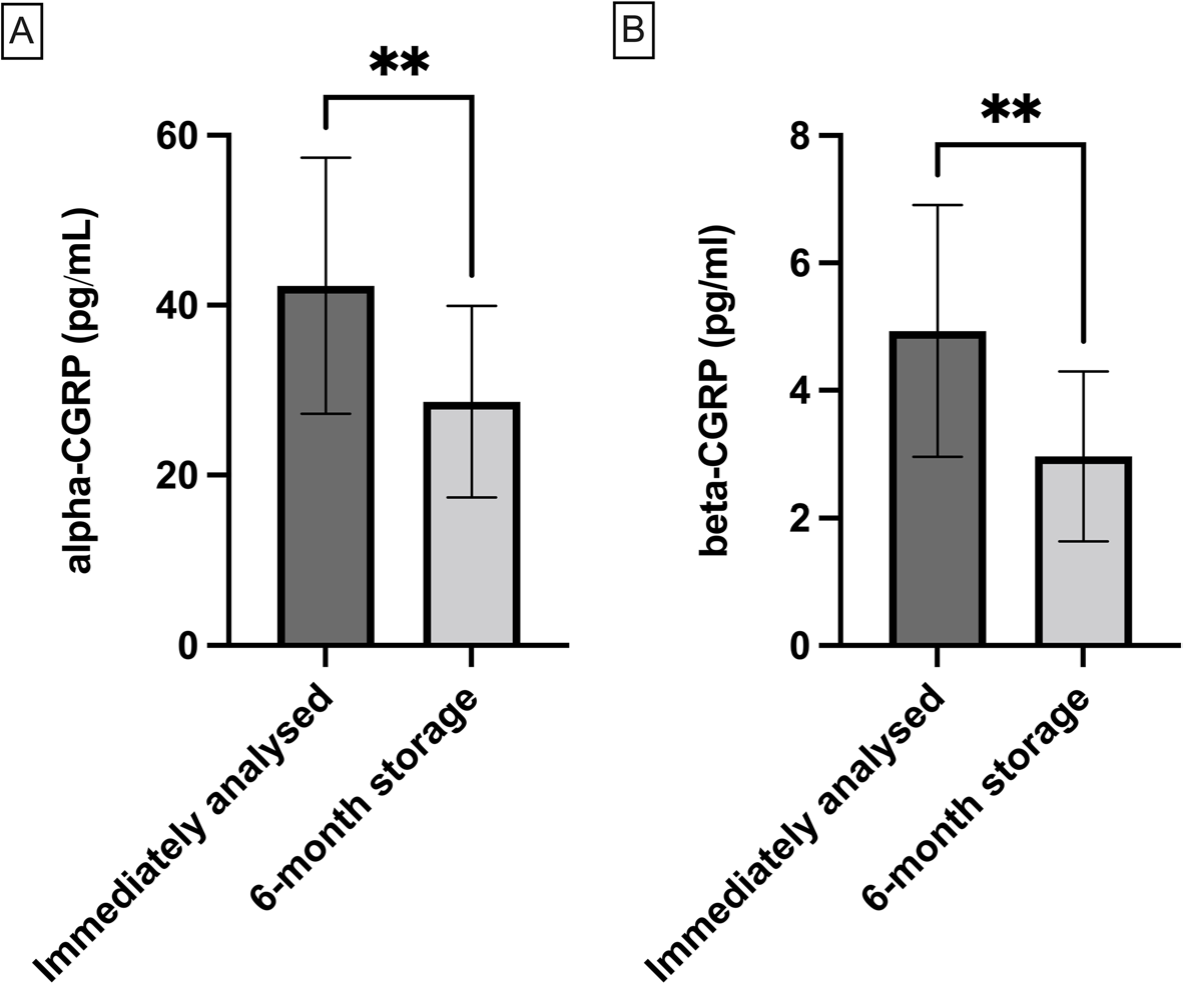
Effect of storage: changes of individual **A** alpha-CGRP and **B** beta-CGRP values when samples were immediately analysed or analysed when they surpassed 6 months storage. Data is shown as average ± SD. Comparisons were made using Wilcoxon matched-pairs signed rank test. **p < 0.01

The first significant differences between samples which were measured before they remained stored at -80°C for a month (alpha: 42.3 ± 15.1pg/mL; beta: 4.9 ± 2.0pg/mL) and assayed after this date appeared from the sixth month of storage for both alpha-CGRP and beta-CGRP (alpha: 28.6 ± 11.3pg/mL, *p* < 0.01; beta: 3.0 ± 1.3pg/mL, *p* < 0.01) (Fig. 3).

### Analysis of our database

**Fig. 4:**
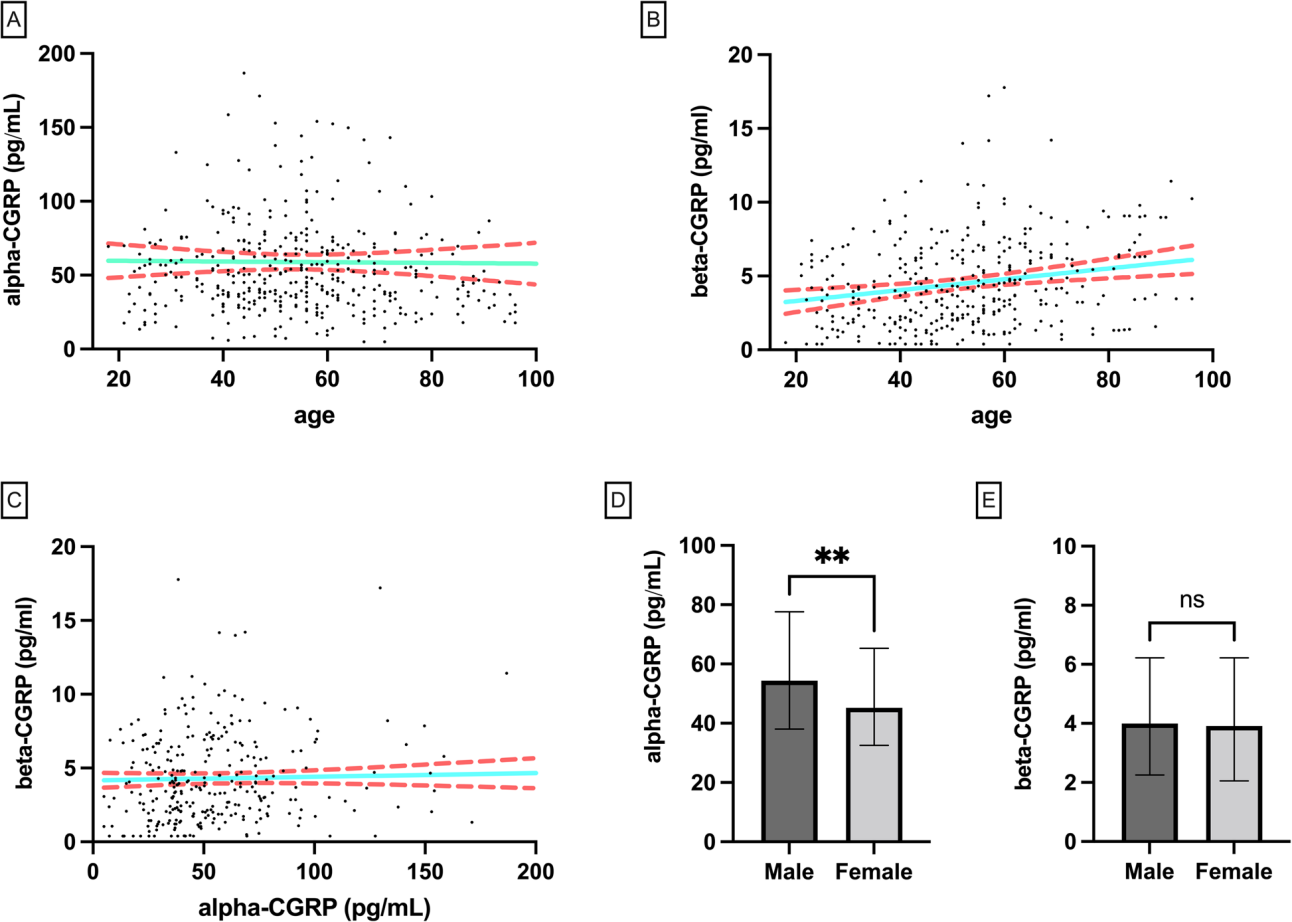
In-house data analysis: **A** distribution of alpha-CGRP levels vs. age, green line represents a linear regression and red dotted line represents the CI; **B** distribution of beta-CGRP levels vs. age, green line represents a linear regression and red dotted line represents the CI; **C** distribution of beta-CGRP vs. alpha-CGRP levels, green line represents a linear regression and red dotted line represents the CI; **D** comparison of alpha-CGRP concentrations in subjects sorted by sex; **E** comparison of alpha-CGRP concentrations in subjects sorted by sex. Data is shown as average ± SD. Comparisons were made using Mann–Whitney U test, ns: non-significant; ** *p* < 0.01

Alpha and beta-CGRP did follow a normal distribution and averaged (median with IQR) 37.5 (28.2–54.4)pg/mL and 4.6 (2.4–6.4)pg/mL, respectively. Spearman correlation between alpha-CGRP and age was non-significant (*p* = 0.300; *r* = -0.05), while it was significant for beta-CGRP and age (*p* < 0.0001; *r* = 0.24). When these correlations were analysed with females and males alone it kept being non-significant for alpha-CGRP (male: *p* = 0.151, *r* = -0.14; female: *p* = 0.514, *r* = -0.04) and significant for beta-CGRP (male: *p* = 0.028, *r* = 0.21; female: *p* < 0.0001, *r* = 0.26). Alpha and beta-CGRP levels did not correlate significantly (*p* = 0.056; *r* = 0.11). When sorted by sex, groups had no significant differences in their age distribution (male: 55.6 ± 17.7 years; female: 54.1 ± 16.9 years; *p* = 0.222), and showed significant differences in their alpha-CGRP content (median [IQR]; males: 54.4 [38.1–77.6] pg/mL; females: 45.2 [32.5–65.3] pg/mL; *p* < 0.01) and unaltered beta-CGRP levels (median [IQR]; males: 4.0 [2.3–6.2] pg/mL; females: 3.9 [2.1–6.1] pg/mL; *p* = 0.728) (Fig. 4).

## Discussion

### Article review

Our literature analysis (Table 1) shows that studies based on CGRP determinations are highly variable in terms of measuring method and study design, including sample source, sample processing, inclusion/exclusion criteria for patients and controls and aim of the study [14, 15, 19, 31, 39, 42, 60, 66, 68]. Data analysis and presentation of laboratory determinations is also changeful, which hinder the comparison of the data. Despite all the difficulties, it results obvious that the overall outcomes and the conclusions drawn from them are inconsistent across works. Some authors have hypothesized that methodological differences might be the reason for such discrepancies [55, 56], and, although this is likely to be the case, there is not to date a consensus of how CGRP determinations should be carried out.

If we analyse the methods used to measure CGRP in migraine patients we can see there have been mainly based on two different techniques, RIA and ELISA. RIA was the first, and until the late 2000s, the only one employed. RIA is based on the competitive incubation for specific antibody sites to form antigen–antibody complexes of radio-labelled and native unlabelled antigen. At equilibrium, the complexes formed are separated from the unbound antigen with a resulting ratio between these two. The bound/free antigen ratio is dependent on the amount of native antigen present in the sample as the radio-labelled is always added at a stable known concentration [72]. Therefore, this technique relies on the antiserum used, which has to provide an appropriate specificity in order to detect the antigen but no other analogues, and a proper affinity to do so in the range of interest.

The use of different antisera across all the CGRP-measuring studies based on RIA is a main source of variability among articles (Table 1). Works employing the same protocol, antiserum, and sample source usually have similar peptide concentrations [14, 39, 47], with some exceptions [48], while the use of different brands containing different antiseras and protocols show differing concentration ranges even when performed with same sample source [15, 39, 63, 64], and even if they were done by the same specialist technician with the same samples [48]. Another problem is that even though studies with the exact same quantification method obtain similar concentration ranges they arrive to clashing conclusions, such as the presence of differences in CGRP concentrations between interictal migraine patients and healthy controls [17, 65].

**Fig. 5:**
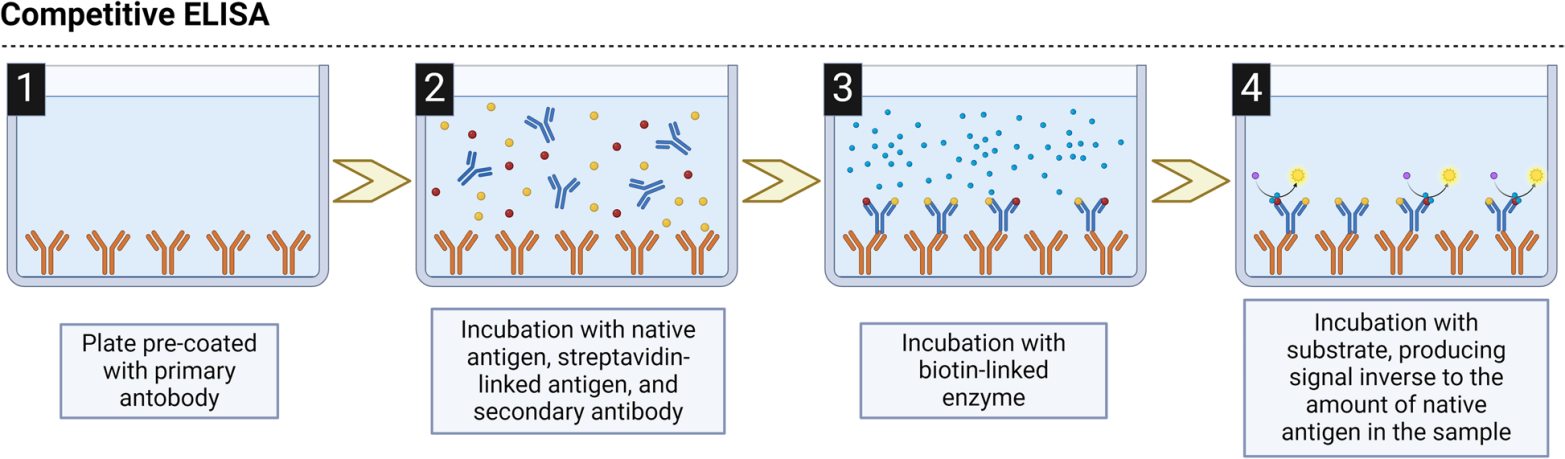
Schematic representation of a competitive ELISA protocol

**Fig. 6:**
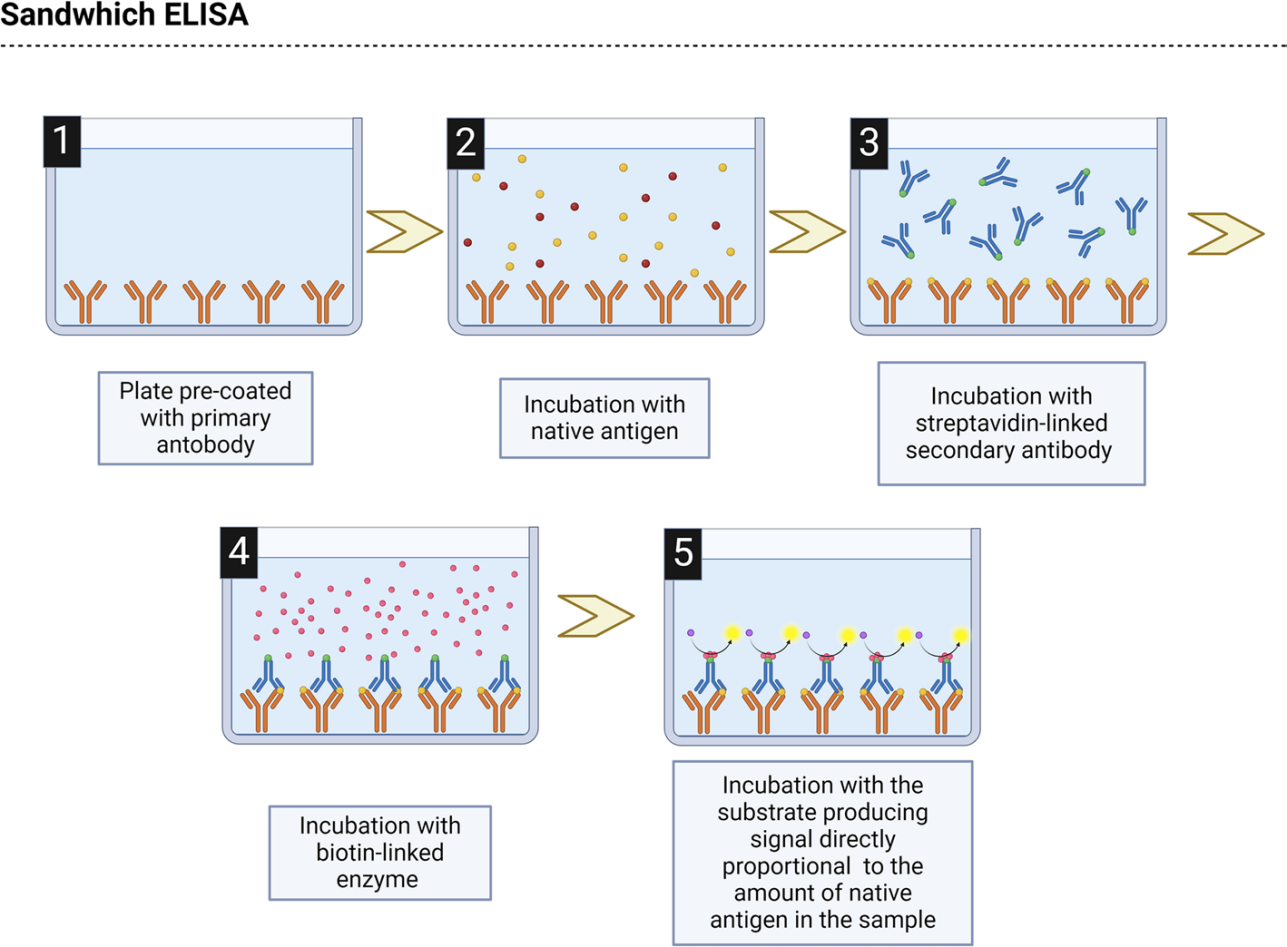
Schematic representation of a sandwich ELISA protocol

ELISA technique first appears to be used to determine CGRP concentration in migraine patients in 2007 [21]. ELISA is an immunological assay based on the interaction between the antigen and a primary antibody against the antigen of interest. These will interact, forming a complex that is later confirmed through the enzyme-linked antibody catalysis of an added substrate, which can be quantitatively measured using readouts from either a luminometer or a spectrophotometer. ELISA techniques are broadly classified into direct, indirect, sandwich, and competitive ELISA. For CGRP determinations only competitive and sandwich ELISA have been employed. Competitive ELISA involves a competition between the sample antigen and the plate-coated antigen for the primary antibody, followed by the binding of enzyme-linked secondary antibodies (Fig. 5). Sandwich ELISA technique includes a sample antigen introduced to the antibody-precoated plate, followed by sequential binding of detection and enzyme-linked secondary antibodies to the recognition sites on the antigen (Fig. 6) [73]. In both cases, and similarly to what has been pointed out for RIA, the techniques rely on the specificity and sensitivity of the antibodies included in the kit. This is the reason why ELISA-based studies are also subjected to the exact same issues associated with RIA-based works. As it has been described, investigations using the same brand also reports similar peptide concentration ranges [25, 26, 30, 44, 49, 67], even though this is not always the case [32], but, most importantly, those using different kits clash in the range of concentrations [23, 61, 62] on top of the conclusions drawn [33, 61]. For this point we need to explain that kits from USCN Life Sciences and Cloud Clone Corp., and from Peninsula Laboratories and BMA Biomedicals have been considered as only two brands since these companies have merged or have been acquired by the other at some point in their history. Moreover, and this last point serves as an example, there is a lack of information by part of the researchers regarding the kits used, because sometimes the brand cited offers more than one kit or two different brands over the history have been in charge of its production, and with the given information it cannot be inferred which one it was [27, 34]. This could be the reason why across studies using kits from the same brand they obtained different concentrations. Also, this lack often comes from the manufacturers, which most of the times do not report essential information to the user such as the specific epitope recognised by the antibodies or their cross-reactivity for analogues of CGRP. This has caused some controversies such as works employing kits specifically designed, according to the manufacturer, for the detection of beta-CGRP reporting results as total-CGRP [35, 36, 59] without proving in their papers whether the technique recognises alpha, beta, or total-CGRP.

CGRP has been analysed in a broad number of samples sources including plasma and serum from the peripheral circulation and jugular vein, CSF, saliva, tear fluid and GCF. Due to the enormous variability of concentrations found within the sources (Table 1) and the fact that results are not homogenous even when the same technique and sample source were used, we thought the comparison between sample sources did not make sense.

Nonetheless, and because our group has focused on the determinations in serum with ELISA, we have done a specific analysis of the studies matching these two criteria. There seems to be a consensus range achieved by most of the studies, independently of the brand employed, and which approximately goes from 15 to 150 pg/mL for total and alpha-CGRP, because the data from the literature exhibits that most of the measured CGRP is the alpha isoform, and from 2 to 10 pg/mL for beta-CGRP.

Because there are examples of different works employing the same method, specific technique, sample source and similar inclusion/exclusion criteria whose results are contradictory [14, 33, 47, 59], we cannot conclude that all the problematic with CGRP measurements is related to the quantifying method and/or the sample chose by the authors. There has to be other factors playing a role in the discrepancies, such as fluctuations with the circadian [74] or with the menstrual cycles [58, 59, 75], effect of resting/exercise [76, 77], fast degradation of the peptide due to its short half-life [78], long-term storage stability [55], migraine and other comorbidities [69, 71, 79–81], and the effects of pharmacological treatments [26, 28, 38–44]. From our review we could not analyse these parameters, because they were not displayed with enough accuracy in most articles.

### Experimental studies

Here, in an effort to provide more detailed information about the suitability of serum from peripheral blood for CGRP determinations, we carried out a series of experiments in order to shed light on some of the main questions regarding the lack of consistency with CGRP quantifications beyond the data already discussed from our review.

#### Kit analysis

We have found that the specific ELISA kit employed has a crucial effect on the CGRP measurements, showing completely different concentration ranges depending on the reference.

Besides the differences in the range we have obtained some alarming results. One of the kits assayed, from Biorbyt, did not meet the reproducibility criteria, which automatically should make this kit unsuitable for any kind of research. On top of that it did not conserve the linearity when diluting the samples which adds more doubts to its reliability. The one from BMA Biomedicals, even though a kit from this brand has been used for a published work when the company had the name Peninsula Laboratories [24], showed for 4 different times results below the detection limit (20pg/mL), contradicting the data of the cited article. Once again, these data call for a more exhaustive description of the methodology, not only by the researchers but also the companies.

The other two kits assayed fulfilled all the quality requirements and presented a range of measurements which fit the range observed in studies using the same sample source. Because the kit from CUSABIO is specific for beta-CGRP we have considered that the objective range for this kind of determination is different to the range for the Abbexa kit, which detects alpha-CGRP. This comes with no surprise because notwithstanding we have not displayed it, in our previous works the exact internal validations were performed and our researches already shown that these kits were reliable and were in accordance with the results published in the past by other groups [25–27, 31, 35, 36, 49].

Overall, the analysis of the kits performed here acts as a probe that the determination methodology needs to be carefully assayed and critically analysed as this is the ultimate guarantee of the validity of the data. Because we have already done so with the 4 kit references listed in this study, we would like to encourage researchers to share their internal validation data with other kits they might have been using, as well as to invite the companies to share more details about their products, as we believe it has been a huge limitation in the field and this would produce a significant advance, saving a lot of time and money to the research.

#### Influence of sample processing time

Throughout the literature many different studies have acknowledged the reported short half-life of CGRP [38, 52, 55, 56, 82] as a main limitation for their works. Still, many fail to describe precisely enough their methodology for sample processing so readers can infer how this limitation took place. This problem has been pointed out before and the latest works have included a more accurate description of the sample processing [35, 36, 38, 55]. To avoid this rapid degradation of the peptide Messlinger et al. [55] proposed buffering the sample with peptidase inhibitor, but they concluded that immediate freezing was the most effective way to preserve CGRP content.

We did not add peptidase inhibitors as we were using serum as sample and the addition of a peptidase inhibitor needed to be done right after centrifugation but we opt to freeze the samples immediately. Our results show that the degradation of the peptide did not happen, at least in the first 24 h, when samples were stored at 4°C. This complies with the instructions of most of the ELISA kits our group has assayed and which provide a window of time for sample storage depending on the temperature, specifying that samples can be stored at 4°C for up to 24 h before being analysed. These data appear to be contradicting the results of Kraenzlin et al. [78] regarding the half-life of CGRP. One could argue that the content of serum and plasma is different and the differences found in these studies could be accounted for the binding of CGRP to cellular compartments or to fibrinogen, effectively modifying its degradation. However, the cited article, performed in 1985, is not exempt from limitations and should be reconsidered when analysing the stability of the peptide, at least in isolated biological fluids. First, this pharmacokinetic (PK) study fails to achieve some critical points that are currently required for this kind of works [83]. CGRP concentration should achieve a steady state in order to be able to extrapolate the half-life as it at this point when the phenomena of absorption, distribution, metabolism and excretion have reached and equilibrium and therefore stopping the infusion will give the information about the actual elimination half-life. Moreover, results from human in-vivo PK studies are not necessarily equivalent to those obtained from in-vitro or animal models in-vivo [84]. Our findings show that serum freezing does not need to be immediate as long as it is kept in the fridge after instant centrifugation following the clotting. This discovery has the potential to ease the methodology of sample processing for CGRP determinations. Although this is a disruptive finding, data should come with no surprise as other neuropeptides with similar and even shorter half-life than CGRP, such as vasoactive intestinal peptide (VIP) [85], amylin [86], and pituitary adenylate cyclase activating peptide-38 (PACAP-38) [87] are being measured without controversy over the sample processing time [33, 51, 88].

#### Long-term storage

Another point recurrently mentioned in the literature is the long-term stability of the molecules when frozen. Available data indicates that storages of 8 months [55] significantly decrease the concentration of CGRP. Our results show that storages over 6 months have a decreasing effect on the serum levels of both isoforms of the peptide. With all the evidences collected future research should specify the time samples remained stored prior to being assayed as this could be a main limitation of the study and to date this data is not usually displayed. This opens up the question about whether controls should be matched not only by age and sex but also by the time their samples remained stored until measured, meaning that both groups, patients and controls, should be enrolled simultaneously to ensure the comparability of their CGRP measurements. This point has already been discussed in studies employing CGRP measurements with controversy results where cases and controls were recruited in two different time frames [89].

#### Exercise

The first potential association between physical exercise and CGRP was described by Wyon et al. [90] with an animal model showing that rats had higher concentrations of CGRP in urine, CSF and serum after 1-h of running. Subsequent studies with more animal models have confirmed this relationship [91, 92]. To date the evidence derived from studies with humans is scarce, with only two works [76, 77]. The first one [76] showed that CGRP increased its concentration in samples collected by microdialysis in 8 individuals who had been subjected to eccentric exercise. In the second, completion of a half marathon produced an immediate CGRP increase dependent on the running intensity in 48 individuals [77].

The relevance for these discoveries in clinical practise is limited because subjects do not usually perform that kind of exercises right before a blood sampling. This is why we analysed the effect of exercise in a way that would reflect more accurately what might be happening at the actual sampling. The results showed that this kind of practise does not have an effect on alpha nor beta-CGRP levels and consequently the patients do not need to be on a strict rest prior to the blood extraction. However, data need to be managed carefully because the exact amount of exercise that has an effect has not been described yet and because the window between the no effect of a 20 min run and a half marathon is wide.

All the results obtained from the experimental analysis would need to be further explored with a bigger number of participants and to be tested in other samples sources that are being considered for CGRP determinations. Nonetheless, it is important to highlight that when considering the future use of CGRP as a biomarker it is necessary to select a sample source that is easy to obtain, which does not have irregular fluctuations associated with unknown factors and which offers reproducible and robust results. Jugular blood, tear fluid, CSF, GCF are not easy to obtain and saliva sampling has to follow very strict protocols to be reliable [93], so our opinion is that future research should perhaps be focusing in plasma and serum from peripheral blood.

### In-house meta-analysis

Our results show that, with a huge number of participants, the levels obtained with Abbexa and CUSABIO kits fit the consensus range seen in the literature review for alpha and beta-CGRP, respectively, and contribute to set a more standardized range of concentration for the peptides.

The effects of sex and age on the circulating levels of CGRP is a point which has not been explored deeply enough. While some studies affirm that CGRP can correlate with age [38], others do not find such correlation [35, 70]. For the data obtained from our in-house samples we found that beta-CGRP correlated positively with age, contradicting previous results obtained with the same kit in plasma and saliva [35]. Besides, the sub-group comprised by males had different alpha-CGRP content that the female, a finding which had not been described. Taking all these data together, the results call for a stricter control of the group design, which would need to be carefully matched in terms of sex and age, to avoid the effect that these two parameters could have on the comparisons.

Also, as the discrimination between alpha and beta-CGRP in research papers has recently begun [38, 69–71], we have shown that these two peptides do not correlate their circulating levels and therefore the results obtained from measuring one or the other are not interchangeable and could lead to opposite conclusions because these molecules can have different behaviours even within the same disorder [38].

### Strengths and limitations

Our work has several strengths. Our literature review summarizes in an easy to understand way all the mess regarding CGRP measurements, showing all the differences not only in terms of results, but also in their aims, design, measuring methodologies and conclusions, allowing for a critical analysis and which will serve as a basis for future comparisons.

Due recent literature has begun to differentiate between alpha and beta-CGRP, we have performed all our experiments to continue doing so, in an effort to expand the knowledge about the different traits of the two molecules.

All the enrolled individuals of the analysis of exercising and duration of the sample processing had their blood extraction performed at the same day and time, limiting the variability that the circadian cycle might have on the levels of the peptide, and all of them were carried out at our laboratory facilities, ensuring an immediate processing and freeze of the serum. Samples for the long-term storage analysis were also obtained at the same time of the day and all of them were collected within a week and assayed for the first and the second time altogether, limiting the effects of different storage time until the determinations and intra-assay variations.

Nonetheless, it has also some limitations that need to be listed. Although we had a bigger list of ELISA kits that have been employed by other researchers, we could not test them all and we decided to probe only 4, including both competitive and sandwich ELISA targeting total, alpha and beta-CGRP. The validity of other kits apart from the ones included in this study would need to be evaluated separately. Also, the results derived from our methodological experiments should be tested in other samples sources as we only included serum because this is, in our opinion, the best sample source for CGRP determinations. For the analysis of our data base, we acknowledge that we did not account for some of the comorbidities of the patients when analysing the effects of sex and age, but because these samples were from our bio-bank their clinical information was limited to the original aim why they were obtained and therefore did not allow such kind of correction.

## Conclusion

We have reviewed the different results obtained throughout the years measuring CGRP making an effort to highlight their differences in terms of aim, inclusion/exclusion criteria, methodology, data display and conclusions. We have also analysed the way these differences might have affect the CGRP levels reported and we have come to the conclusion that is not only the sample or the method (RIA or ELISA) but even the brand employed which ultimately determine the concentration range.

Finally, we have illustrated some new features of CGRP determinations in serum which are very valuable for the planning of future studies. Concentrations of alpha-CGRP and beta-CGRP seems to be about (median with IQR) 37–5 (28.2–54.4) pg/mL and 4.6 (2.4–6.4) pg/mL, respectively, according to our in-house analysis, which agrees with what can be seen from the literature review. The facts that serum kept refrigerated conserves the CGRP content up to 24 h and that moderate exercise does not exert a modulation effect on the concentrations will ease the design of sample extraction and processing protocols. Also, we point out that storage time should be controlled as a new way to ensure the validity of results, probably by the simultaneous enrolling of all the subjects included in the study and/or by assaying their samples within similar time-ranges from the extraction. Ultimately, we have shown that alpha and beta-CGRP should be analysed separately as the isoforms do not correlate their concentrations and it has been illustrated in the literature that these can have different behaviours within the same disorder.

Overall, this work has brought new methodological data to progress in our way to evaluate the actual role of CGRP as a migraine biomarker at the same time it has evaluated the previous advances with a critical point of view, trying to produce a constructive criticism that will help to progress in this challenging topic.

## Data Availability

No datasets were generated or analysed during the current study.

## Abbreviations

BCA: Bicinchoninic acid protein assay
CDH: Chronic daily headache
CGRP: Calcitonin gene-related peptide
CI: Confidence interval
CM: Chronic migraine
COC: Combined contraception
CSF: Cerebrospinal fluid
CV: Cubital vein
EIA: Enzyme immune assay
ELISA: Enzyme-linked immunosorbent assay
EM: Episodic migraine
GCF: Gingival crevicular fluid
HC: Healthy controls
ICHD-3: International classification of headache disorders 3rd edition
IQR: Interquartile range
JV: Jugular vein
MA: Migraine with aura
MO: Medication overuse
MWA: Migraine without aura
PACAP-38: Pituitary adenylate cyclase activating peptide-38
PK: Pharmacokinetics
PM: Post menopause
RIA: Radioimmuno assay
RMC: Regular menstrual cycle
SD: Standard deviation
SEM: Standard error of mean
VIP: Vasoactive intestinal peptide
WA: Without aura
